# The role of RNA modifications in cancer translational control

**DOI:** 10.1080/15476286.2026.2694854

**Published:** 2026-06-24

**Authors:** Merin Joy, Alice Cleynen, Nikolay E. Shirokikh

**Affiliations:** a Children’s Medical Research Institute, Westmead, NSW, Australia; b Institut Montpelliérain Alexander Grothendieck, Université de Montpellier, CNRS, Montpellier, France; c IRL FAMSI, CNRS International Research Laboratory, Australian National University, Canberra, Australia; d Australian Centre for RNA Therapeutics in Cancer, School of Human Sciences, University of Western Australia, Perth, WA, Australia

**Keywords:** RNA modifications, epitranscriptomics, cancer translation, m6A, ribosome heterogeneity, translational control

## Abstract

RNA modifications have emerged as central regulators of cancer translational control. Unlike transcriptional reprogramming, which unfolds over hours, modification-dependent translational rewiring enables rapid proteomic adaptation to the nutrient-deprived, hypoxic, and immunologically hostile tumour microenvironment. Yet most existing reviews organize epitranscriptomic mechanisms by modification type or cancer hallmark, obscuring the mechanistic logic by which chemical marks collectively reshape the translational apparatus. This review adopts a translation-centric framework, examining how the most abundant modifications on mRNAs, tRNAs, and rRNAs regulate each stage of protein synthesis in malignant cells. We survey the epitranscriptomic toolkit, including modification chemistries, enzymatic writers, readers, and erasers, and detection technologies including nanopore direct RNA sequencing. We then trace how modifications control initiation (m6A-driven mRNA circularization, cap-independent translation *via* eIF3 and eIF4G2, rRNA 2′-O-methylation-directed cap-to-IRES switching), elongation (m6A-induced ribosome stalling coupled to mRNA decay, tRNA mcm5s2U-mediated codon-biased translation, YTHDF1-dependent elongation factor recruitment), and termination (pseudouridine-mediated stop codon readthrough, NMD evasion). Crucially, we show that mRNA, tRNA, and rRNA modifications do not act in isolation but form integrated networks. For example, mRNA m6A and tRNA mcm5s2U operate on opposing arms of the same regulatory axis, with direct implications for therapeutic design. We assess the expanding drug pipeline, from the METTL3 inhibitor STC-15 now in Phase 1b/2 trials and METTL3-targeting PROTACs to FTO and ADAR1 inhibitors, and argue that biology-informed combination strategies targeting multiple modification axes will be essential for durable clinical responses.

## GRAPHICAL ABSTRACT

**Figure:**
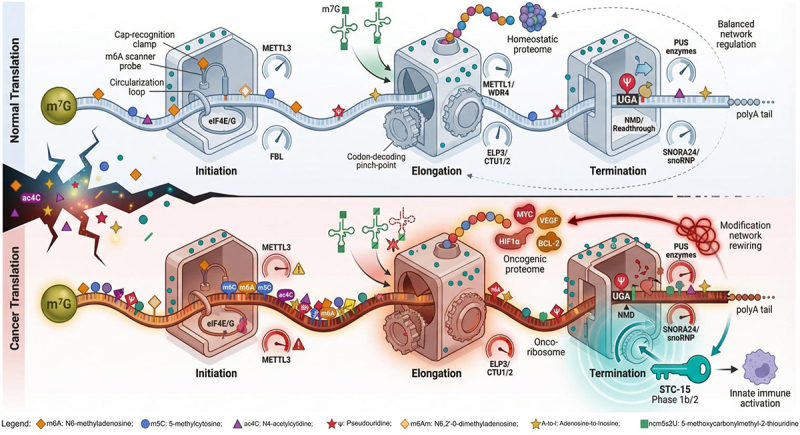

## Introduction

### RNA modifications: from discovery to translational relevance

The discovery of pseudouridine in yeast transfer RNA in 1957 marked the first recognition that RNA nucleotides could be chemically altered beyond their canonical forms [1]. Over the following decades, additional modifications were identified across all RNA species, such as 5-methylcytosine (m5C) in ribosomal RNA during the 1960s, N6-methyladenosine (m6A) in messenger RNA in the 1970s, yet their functional significance remained largely unexplored [2,3]. The field experienced a renaissance in 2012, when two landmark studies mapped m6A across the mammalian transcriptome at high resolution, revealing that RNA modifications were not merely structural relics but dynamic, reversible regulators of gene expression [4,5]. This catalysed the emergence of epitranscriptomics as a discipline, and to date more than 170 chemically distinct RNA modifications have been catalogued [6].

The therapeutic potential of manipulating RNA modifications became broadly apparent during the COVID-19 pandemic, when N1-methylpseudouridine (m1Psi) proved essential for the efficacy of mRNA vaccines by reducing immunogenicity while sustaining translational output [7,8]. More recently, the first small-molecule inhibitor targeting an RNA-modifying enzyme – STC-15, directed against the m6A methyltransferase METTL3 – entered clinical trials for advanced solid tumours, with Phase 1b/2 expansion cohorts now recruiting patients across four cancer indications [9,10]. These developments underscore that RNA modifications have transitioned from biochemical curiosities to actionable targets in oncology.

### Cancer translation as a regulatory vulnerability

Translation represents the most energy-intensive process in dividing cells, consuming a substantial fraction of cellular ATP and GTP to sustain the protein synthesis demands of proliferation [11,12]. Cancer cells are particularly dependent on translational control: unlike transcriptional reprogramming, which unfolds over hours, translational regulation enables rapid proteomic adaptation within minutes, a critical advantage for tumour cells navigating the nutrient-deprived, hypoxic, and immunologically hostile tumour microenvironment [13,14].

RNA modifications have emerged as central to this translational plasticity. Chemical marks deposited on mRNAs, transfer RNAs, and ribosomal RNAs collectively reprogram every stage of translation. from the recruitment of ribosomes to mRNA, through the decoding of codons, to the recognition of stop signals, and thus enabling cancer cells to selectively synthesize oncogenic proteins while suppressing tumour-suppressive programmes [15,16]. The reversible, dynamic nature of these modifications provides a layer of regulation that is fundamentally distinct from genetic mutation: it is rapid, tuneable, and responsive to microenvironmental cues, making it both a driver of malignant adaptation and a therapeutic vulnerability.

Recent years have seen an explosion of reviews cataloguing individual RNA modifications and their associations with specific cancer types [17,18]. However, most existing reviews organize their content by modification type (m6A, m5C, pseudouridine, *etc*.) or by cancer hallmark (proliferation, metastasis, immune evasion), which, while informative, obscures the mechanistic logic by which modifications collectively reshape the translational apparatus. A framework centred on the translation cycle itself and examining how modifications regulate initiation, elongation, termination, and ribosome quality control is lacking but essential for understanding how epitranscriptomic dysregulation drives oncogenic protein synthesis and for identifying rational therapeutic strategies.

### Scope and organisation of this review

This review addresses this gap by examining RNA modifications through the lens of translational control in cancer. Rather than providing an exhaustive catalogue of every modification-cancer association, we focus on the mechanistic principles by which chemical marks on mRNAs, tRNAs, and rRNAs regulate each stage of protein synthesis in malignant cells.

We begin with an overview of the epitranscriptomic toolkit, including the major modifications, their enzymatic regulators, and current detection technologies (The Epitranscriptomic Toolkit). We then present a mechanistic framework of the translation cycle, annotated with the sites at which RNA modifications exert regulatory control, encompassing both cytoplasmic and mitochondrial translation (Translation and RNA Modifications: A Mechanistic Framework). The core of the review examines modification-dependent mechanisms at each translational stage: initiation (Initiation Control by RNA Modifications), elongation (Elongation Control by RNA Modifications), and termination and ribosome quality control (Termination and Quality Control: RNA Modifications at the End of the Line). We subsequently explore the emerging understanding of integrated modification networks and their combinatorial effects on translation (Integrated Modification Networks: How Multi-Layered Epitranscriptomic Crosstalk Shapes Malignant Translation). Finally, we assess the therapeutic landscape, including small-molecule inhibitors in clinical development, combination strategies, and biomarker applications (Therapeutic Targeting of RNA Modifications in Cancer Translation), before discussing future directions and concluding perspectives (Future Directions and Concluding Perspectives).

By adopting this translation-centric framework, we aim to illuminate the mechanistic logic that connects epitranscriptomic dysregulation to oncogenic protein synthesis, and to identify the most promising avenues for therapeutic intervention in this rapidly advancing field.

## The epitranscriptomic toolkit

### Chemical diversity of RNA modifications in cancer translation

**Figure 1.**
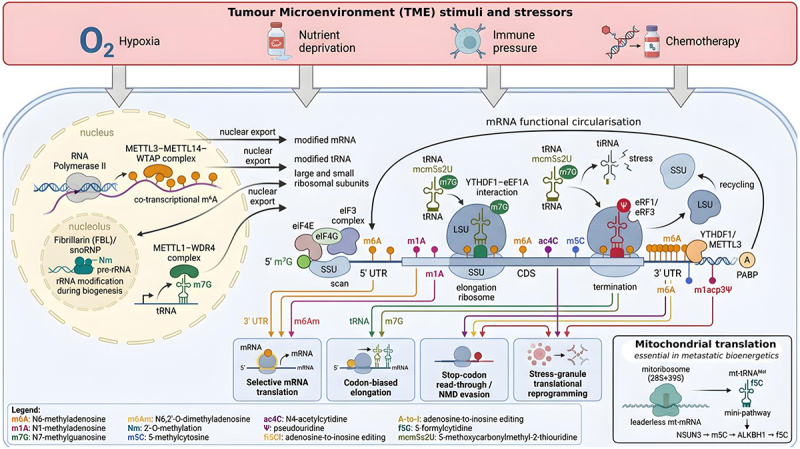
RNA modification landscape in cancer translation. Overview of the major RNA modifications that regulate mRNA fate and protein synthesis in malignant cells. Modifications are positioned according to their primary RNA substrates (mRNA, tRNA, rRNA) and the translational stages they influence (initiation, elongation, termination, and quality control). External stimuli from the tumour microenvironment, including hypoxia, nutrient deprivation, and immune pressure, alter the activity of modification enzymes, reprogramming the epitranscriptomic landscape to favour oncogenic translation. Key modifications shown include m6A, m7G (cap and internal), m5C, ac4C, ψ, 2′-O-methylation (Nm), and wobble-position tRNA modifications (mcm5s2U, f5C). Arrows indicate functional connections between modification events and translational outcomes, including selective mRNA translation, codon-biased elongation, stop-codon read-through, and stress-granule-associated translational reprogramming.

More than 170 chemically distinct modifications have been identified across all RNA classes, ranging from simple methyl additions to complex multi-step transformations [6]. Although many of these remain poorly characterized, a subset has emerged as functionally relevant to translational control in cancer (Figure 1, Table 1). These modifications can be grouped by their chemical nature, such as methylation, acetylation, isomerization, deamination, and oxidation, with each type imposing distinct structural and functional consequences on the modified RNA.

**Table 1.** Key RNA modifications in cancer translational control.

Modification	Chemical change	Primary RNA substrate(s)	Writer(s)	Reader(s)	Eraser(s)	Translational role in cancer	Key reference(s)
m6A	N6-methyl on adenosine	mRNA (3′UTR, CDS, 5′UTR)	METTL3-METTL14/WTAP complex	YTHDF1 (translation ↑), YTHDF2 (decay), YTHDF3, IGF2BPs (stability ↑)	FTO, ALKBH5	3′UTR: mRNA circularisation → enhanced initiation; 5′UTR: eIF3-mediated cap-independent initiation; CDS: ribosome stalling → collisions → coupled mRNA decay; Modulated by tRNA mcm5s2U	[4,41]
m5C	C5-methyl on cytidine	mRNA, tRNA, rRNA	NSUN1-7, DNMT2	ALYREF (export), YBX1 (stability)	TET1/2/3 (→hm5C)	mRNA export and stability; co-occurs non-randomly with m6A on same molecules; tRNA structural stability	[22,54]
m1A	N1-methyl on adenosine	mRNA, tRNA	TRMT6-TRMT61A (mRNA), TRMT10C (mt-tRNA)	–	ALKBH1, ALKBH3	Disrupts Watson-Crick pairing → alters RNA structure; cooperates with m6A to accelerate mRNA decay *via* HRSP12-YTHDF2	[20,59]
m7G	N7-methyl on guanosine	mRNA (5′ cap), tRNA (internal pos. 46)	RNMT-RAM (cap); METTL1-WDR4 (internal tRNA)	eIF4E (cap)	DCP2 (decapping)	Cap m7G: essential for cap-dependent translation; Internal tRNA m7G46: stabilises tRNA tertiary structure → codon-biased translation of AGA-enriched cell cycle mRNAs	[21]
Ψ (pseudouridine)	C5-glycoside isomer of uridine	mRNA, tRNA, rRNA	PUS1-10 (standalone); Dyskerin/H/ACA snoRNPs (guided)	–	None identified	Stop codon Ψ: suppresses termination → readthrough products; rRNA Ψ: ribosome decoding fidelity; Amino acid misincorporation at specific codon positions	[31,60]
m1acp3Ψ	Hypermodified pseudouridine (three-step)	18S rRNA (pos. 1248)	SNORA13 (Ψ) → EMG1 (m1) → TSR3 (acp3)	–	None identified	Peptidyl decoding site fidelity; loss in up to 45.9% of CRC → “onco-ribosomes” with skewed translational signature	[61]
2′-O-Me (Nm)	2′-O-methylation on ribose	rRNA, tRNA, snRNA	Fibrillarin/C/D box snoRNPs	–	None identified	rRNA: ribosome fidelity, cap-to-IRES initiation switch; Fractionally methylated sites act as tunable regulatory switches; FBL overexpression → IRES-dependent oncogene translation	[25,26]
ac4C	N4-acetyl on cytidine	mRNA, tRNA, rRNA	NAT10 (sole writer)	–	None identified	Enhances mRNA stability and translation efficiency; promotes cell-cycle progression and drug resistance; acetylates tRNA and 18S rRNA	[27]
mcm5s2U / mcm5U	Wobble uridine modifications	tRNA (anticodon pos. 34)	ELP1-6 complex, CTU1/CTU2	–	None identified	Counteracts m6A-induced codon de-optimisation → stabilises m6A-marked oncogenic mRNAs; Drives codon-biased translation of HIF-1α, glycolytic enzymes, DEK; High mcm5s2U → poor prognosis	[62,63]
f5C	5-formylcytidine	mt-tRNAMet (wobble pos.)	NSUN3 (m5C) → ALKBH1 (oxidation to f5C)	–	–	Enables decoding of both AUG and AUA as methionine in mitochondria; NSUN3 loss abolishes metastatic competence in CD36+ tumour-initiating cells	[64]
A-to-I (inosine)	Deamination of adenosine	mRNA (coding and non-coding), dsRNA	ADAR1 (p150, p110), ADAR2	–	–	Recodes A as G during translation → altered protein function; ADAR1 editing suppresses MDA5/PKR sensing → immune evasion, prevents PKR-mediated translational shutdown	[32,33]
m6Am	N6-methyl at first transcribed nucleotide	mRNA (cap-adjacent)	PCIF1	–	FTO	Confers resistance to decapping → stabilises mRNA subset; altered expression in cancer	[24]

Summary of the major RNA modifications relevant to cancer translational control discussed in this review. For each modification, the chemical nature, primary RNA substrates, enzymatic regulators (writers, readers, erasers), translational function in cancer, the section(s) in which it is discussed, and key references are indicated. Dashes (-) indicate that no dedicated reader or eraser has been identified for that modification. Modifications are listed in approximate order of coverage within the review. Abbreviations: CDS, coding sequence; CRC, colorectal carcinoma; IRES, internal ribosome entry site; mt, mitochondrial; UTR, untranslated region.

Methylation modifications constitute the largest and most intensively studied group. N6-methyladenosine (m6A) is the most abundant internal modification on mRNA, enriched in 3′ untranslated regions (UTRs) and near stop codons, where it regulates mRNA stability, nuclear export, and, most pertinently to this review, translational efficiency through recruitment of specific reader proteins [4,5]. Critically, m6A maintains Watson-Crick base-pairing capacity, meaning its effects on translation are mediated primarily through altered protein-RNA interactions rather than disrupted coding potential [19]. By contrast, N1-methyladenosine (m1A) introduces steric clashes that disrupt Watson-Crick pairing, destabilizing RNA secondary structures and thereby altering translational dynamics in a position-dependent manner [20]. N7-methylguanosine (m7G) is essential at the 5′ cap of all mRNAs for cap-dependent translation, but internal m7G deposited in tRNAs by the METTL1-WDR4 complex has gained prominence for its role in oncogenic transformation: elevated METTL1 drives enhanced translation of cell-cycle genes through tRNA stabilization and correlates with poor survival across multiple cancer types [21. 5]-Methylcytosine (m5C), deposited on mRNAs, tRNAs, and rRNAs by NSUN-family methyltransferases and DNMT2, enhances RNA stability and modulates nuclear export; its oxidation product 5-hydroxymethylcytosine (hm5C), generated by TET enzymes, represents a functionally distinct mark with emerging roles in cancer gene regulation [22,23]. N6,2′-O-dimethyladenosine (m6Am), occurring at the first transcribed nucleotide adjacent to the mRNA cap, is installed by PCIF1 and confers resistance to decapping, stabilizing a subset of mRNAs whose translational output becomes altered in cancer [24]. Finally, 2′-O-methylation (Nm), deposited on rRNAs and tRNAs by fibrillarin guided by C/D-box small nucleolar RNPs, influences ribosome fidelity and the balance between cap-dependent and IRES-dependent translation, a balance frequently tipped towards oncogenic programmes in cancer cells [25,26].

Beyond methylation, several other chemical classes of modification are important for cancer translation. N4-acetylcytidine (ac4C), catalysed exclusively by the acetyltransferase NAT10 using acetyl-CoA as a donor, enhances both mRNA stability and translation efficiency. ac4C modifies not only tRNAs and rRNAs but also mRNAs and primary microRNAs, and recent work implicates it in cell cycle regulation, drug resistance, and cancer metabolism [27,28]. Pseudouridine (Ψ), the C5-glycoside isomer of uridine and the most abundant RNA modification by mass, arises from rotation around the glycosidic bond and gains an additional hydrogen bond donor at the non-Watson-Crick edge [29]. Ψ stabilizes RNA duplexes and, when present at stop codons, can suppress translation termination, a property with profound implications for quality control evasion in cancer [30,31]. Adenosine-to-inosine (A-to-I) editing, catalysed by ADAR enzymes, effectively recodes adenosine as guanosine during translation, expanding transcriptome diversity. A-to-I editing is dysregulated in numerous cancers, where it contributes to immune evasion, drug resistance, and altered protein function [32,33].

The translational consequences of these non-m6A marks are, in general, less firmly established than for m6A, and we summarize them here with corresponding caution. For m5C, NSUN2-deposited marks in coding sequences and untranslated regions have been reported to influence elongation and ribosome occupancy and to be interpreted by readers such as ALYREF and YBX1, although the magnitude and direction of the translational effects remain debated and some mRNA-level m5C maps have been contested on technical grounds. m1A, by disrupting Watson-Crick pairing in a strongly position-dependent manner, can impede decoding and additionally cooperates with m6A to accelerate transcript decay (see ‘Integrated Modification Networks’), though its abundance and distribution in mRNA are likewise debated. ac4C, deposited solely by NAT10, has been associated with enhanced mRNA stability and translation efficiency across tRNA, rRNA and mRNA substrates, but the stoichiometry and transcript selectivity of mRNA ac4C in cancer require further validation.

The chemical logic connecting modification structure to translational outcome follows several general principles that recur throughout this review. Modifications that alter the Watson-Crick edge (m1A, inosine) directly change coding potential or secondary structure. Those that modify the Hoogsteen or sugar edge (m6A, Ψ, Nm) primarily modulate protein-RNA interactions or RNA stability without disrupting base-pairing. And modifications at the wobble position of tRNA anticodons (mcm5U, mcm5s2U, f5C, m7G at position 46) tune codon-anticodon recognition, thereby shaping codon-biased translation programmes that cancer cells exploit for selective synthesis of oncogenic proteins.

### Writers, readers, and erasers: dynamic regulation of the epitranscriptome

**Figure 2.**
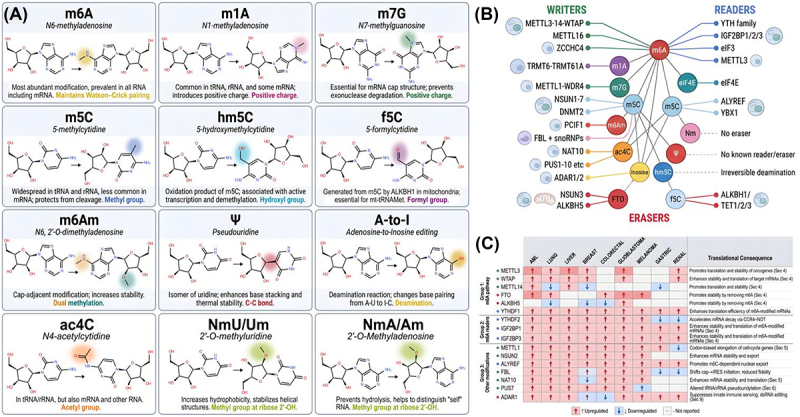
The epitranscriptomic toolkit: writers, readers, and erasers regulating cancer translation. (A) Chemical structures of the major RNA modifications discussed in this review. For each modification, the modified nucleoside is shown with the chemical change highlighted. Modifications are grouped as: purine methylation (m6A, m1A, m7G), cytidine modifications (m5C, hm5C, f5C), cap-adjacent and sugar methylation (m6Am, Nm), isomerisation (ψ), deamination (A-to-I/inosine), and acetylation (ac4C). (B) Writer-reader-eraser network. Writer enzymes (green) that deposit each modification, reader proteins (blue) that interpret the mark, and eraser enzymes (red) that remove it are connected to their cognate modifications. Annotations indicate modifications for which no dedicated reader or eraser has been identified, and modifications whose removal is irreversible (A-to-I editing). (C) Cancer-relevant dysregulation heatmap. Upregulated (↑, red) or downregulated (↓, blue) enzymes across nine common cancer types are shown, grouped by the m6A pathway, m6A readers, and other modification enzymes. The rightmost column indicates the translational consequence of each enzyme’s dysregulation, with cross-references to the review sections where each mechanism is discussed in detail.

RNA modifications are installed, interpreted, and removed by dedicated enzymatic machineries termed writers, readers, and erasers, whose dysregulation in cancer rewires translational programmes (Figure 2) [16].

Writers deposit modifications through diverse catalytic mechanisms. The best-characterized methyltransferase complex comprises METTL3 (the catalytic subunit) and METTL14 (an allosteric activator), together with WTAP, RBM15, and VIRMA, which determine substrate specificity and direct co-transcriptional m6A deposition predominantly in nuclear speckles [34,35]. Beyond this core complex, two additional m6A methyltransferases operate on distinct substrates: METTL16 methylates U6 snRNA and the MAT2A mRNA encoding S-adenosylmethionine synthetase, creating a metabolic feedback loop that couples cellular SAM availability to m6A deposition [36], while ZCCHC4 instals m6A at position A4220 of 28S rRNA in the nucleolus, directly modifying the translational machinery [37] (Figure 2b).The METTL1-WDR4 complex instals internal m7G in tRNAs, directly coupling tRNA modification status to oncogenic gene expression [21]. NSUN-family proteins (NSUN1-7) and DNMT2 catalyse m5C formation across RNA species, with NSUN2 targeting mRNAs and tRNAs while NSUN3 deposits m5C on mitochondrial tRNAMet as the first step towards the essential wobble position f5C modification [38,39]. NAT10, the sole ac4C writer, utilizes acetyl-CoA and ATP hydrolysis to acetylate cytidines in tRNAs, rRNAs, and mRNAs [27]. Pseudouridine is installed by two mechanistically distinct systems: standalone PUS-family enzymes that recognize substrate RNA directly, and H/ACA-box snoRNP complexes in which the catalytic protein dyskerin is guided to specific rRNA sites by snoRNA base-pairing [40]. ADAR1 and ADAR2 catalyse A-to-I editing through hydrolytic deamination, with ADAR1 additionally serving as a key regulator of innate immune signalling through its capacity to edit endogenous double-stranded RNA [32].

Readers translate chemical marks into biological outcomes. The YTH-domain family (YTHDF1-3 and YTHDC1-2) constitutes the primary m6A-reading machinery: YTHDF1 promotes translation through eIF3 recruitment, YTHDF2 targets transcripts for degradation, YTHDF3 functions cooperatively with or independently of YTHDF1 depending on tumour context, YTHDC1 regulates nuclear processing and export, and YTHDC2 (an RNA helicase) resolves secondary structures in 5′UTRs to facilitate ribosome scanning [41,42]. The insulin-like growth factor 2 mRNA-binding proteins (IGF2BP1-3) represent a second class of m6A readers that primarily stabilize target transcripts and enhance their translation [43]. For m5C, ALYREF and YBX1 act as readers that influence nuclear export and mRNA stability, respectively [22]. Notably, some writers also function as readers: METTL3 itself can directly recruit eIF3 to m6A-modified mRNAs, enhancing translation through a mechanism independent of YTH-domain proteins [44].

The functional assignment of the cytoplasmic YTHDF paralogues is itself a matter of active debate. The widely adopted ‘division-of-labour’ model, in which YTHDF1, YTHDF2 and YTHDF3 perform distinct functions in translation, decay and cooperative regulation respectively, has been challenged by a ‘unified’ model proposing that all three paralogues bind the same m6A sites largely redundantly and act principally to promote mRNA destabilization [45]. The discrepancy is attributable in part to differences between transient-overexpression and knockout/knockdown approaches, cell-type context, and the relative paralogue abundances. The translation-promoting and elongation-factor-recruiting activities of YTHDF1/YTHDF3 discussed below are best supported in specific cancer contexts and should not be assumed to generalize; readers should interpret paralogue-specific claims, including those we cite, with this unresolved controversy in mind.

Erasers provide dynamic reversibility. The AlkB-family demethylases FTO and ALKBH5 remove m6A, with FTO also capable of demethylating m6 Am and m1A depending on its subcellular localization [46,47]. ALKBH1 and ALKBH3 target m1A, with ALKBH1 additionally serving as the oxidase that converts m5C to f5C in mitochondrial tRNAMet [39]. TET enzymes oxidize m5C to hm5C and further derivatives, effectively erasing the methyl mark. Importantly, for several modifications, including Ψ, ac4C, and Nm, no dedicated erasers have yet been identified, suggesting that these marks may be regulated primarily through RNA turnover rather than active removal.

The spatial organization of these enzymes within the cell adds a further dimension of regulation. Writers such as the METTL3-METTL14 complex and dyskerin-containing snoRNPs are predominantly nuclear, coupling modification deposition to transcription and ribosome biogenesis. By contrast, readers and erasers frequently operate in the cytoplasm, where they modulate mRNA translation and decay. In cancer, this compartmentalization is often disrupted: cytoplasmic re-localization of METTL3, for example, enables post-transcriptional m6A deposition that enhances translation of oncogenic mRNAs, while nuclear retention of FTO alters the balance of m6A in nascent transcripts [44].

### Detection technologies: a brief overview

No single technology is sufficient for comprehensive epitranscriptomic characterization, and the most rigorous studies integrate multiple orthogonal approaches. The current epitranscriptomic toolkit relies on four complementary detection strategies, each with characteristic trade-offs. Antibody-based immunoprecipitation methods such as MeRIP-seq provide transcriptome-wide coverage but are limited to ~100–200 nucleotide resolution and susceptible to antibody cross-reactivity [4]. Chemical and enzymatic approaches, including bisulphite sequencing for m5C, DART-seq for m6A, and CMC-based methods for Ψ, can achieve single-nucleotide resolution but require specialized protocols [30,48]. Direct detection by liquid chromatography-tandem mass spectrometry (LC-MS/MS) provides unambiguous chemical identification and quantification, while nanopore sequencing now enables simultaneous, single-molecule detection of multiple modifications without chemical pre-treatment, offering a promising platform for mapping co-occurring modifications on individual transcripts [49–51]. Direct RNA sequencing (DRS) enabled by nanopore technology is developing rapidly, with a growing number of RNA modification models now included in default analytical software [49,52,53]. DRS has the potential to provide a single-molecule, single-nucleotide simultaneous detection of various modification types, enabling combinatorial analytical and diagnostic opportunities not possible with the other approaches [54,55]. Computational prediction methods, increasingly powered by deep learning, complement these experimental approaches by identifying candidate modification sites from sequence and structural features, though their accuracy varies by modification type and remains dependent on training data quality [56].

These advantages are counterbalanced by substantial, and currently unresolved, limitations. Modification-calling accuracy varies markedly between modification types and between tools, and per-site false-positive and false-negative rates can be high, particularly for modifications other than m6A and for sites in low-coverage or low-abundance transcripts. Most basecallers infer modifications indirectly from perturbed ionic-current signals using supervised deep-learning models, so their performance is bounded by the quality, balance and representativeness of the training data and degrades on sequence contexts or modification combinations not seen during training. Absolute stoichiometry estimation remains especially challenging and is sensitive to model calibration. Independent benchmarking efforts continue to report limited concordance between methods and emphasize the need for orthogonal validation and standardized reference materials before DRS-derived modification maps, especially the single-molecule co-occurrence calls, can be treated as quantitative ground truth, particularly in a clinical setting [53,57].

Reproducibility is a recognized concern across the field. Antibody-based methods such as MeRIP-seq/m6A-seq, which underpin a large fraction of the cancer m6A literature, are limited not only in resolution (~100–200 nt) but also by antibody cross-reactivity, sensitivity to input amount and immunoprecipitation conditions, and limited concordance between laboratories and analytical pipelines. Benchmarking has shown that apparent differential-methylation signals are often poorly reproducible and can be dominated by technical rather than biological variation [58]. Many cancer association studies rely on a single mapping method without orthogonal validation, and this should be borne in mind when interpreting the magnitude and specificity of reported modification changes.

## Translation and RNA modifications: a mechanistic framework

### RNA modifications as translational switches in cancer

The epitranscriptomic toolkit described above equips cells with a diverse palette of chemical marks, but the critical question for cancer biology is how these marks converge on protein synthesis. Translation is the most energy-intensive biosynthetic process in dividing cells, consuming up to 75% of total cellular energy expenditure, and cancer cells are disproportionately dependent on it to sustain their proliferative, invasive, and stress-adaptive programmes [11,12]. Crucially, translational control operates on a timescale of minutes (far faster than transcriptional reprogramming) granting tumour cells the agility to adjust their proteome in response to hypoxia, nutrient fluctuation, immune attack, or drug exposure [13,14]. RNA modifications sit at the interface of these two properties: they are themselves rapid and reversible, and they act directly on the translational machinery. This convergence makes modification-dependent translational control a uniquely potent axis for oncogenic adaptation, and a correspondingly attractive target for therapeutic intervention.

### General principles: how chemical marks reshape protein synthesis

Before examining individual translational stages, it is useful to outline the general mechanistic principles by which RNA modifications influence translation. These principles are recurring themes throughout this review and fall into four broad categories.

First, modifications alter RNA structure and thermodynamic stability. Pseudouridine rigidifies the sugar-phosphate backbone and strengthens base stacking, stabilizing local helices [65]. Conversely, m1A introduces steric clashes that disrupt Watson-Crick pairing and can melt inhibitory secondary structures in 5′UTRs, facilitating ribosome scanning [20. 2]′-O-methylation (Nm) enhances duplex stability and nuclease resistance, properties that contribute to rRNA structural integrity and tRNA longevity [26].

Second, modifications create, mask, or remodel protein-RNA interaction surfaces. m6A, the most abundant internal mRNA modification, is primarily interpreted through reader proteins, such as the YTH-domain family and IGF2BPs, that recruit translational activators or repressors to modified transcripts [41,43]. Importantly, some modifications function by eliminating binding sites: m6A destabilizes local RNA duplexes through an ‘m6A switch’ mechanism, exposing single-stranded motifs that recruit RNA-binding proteins otherwise occluded by secondary structure [66].

Third, modifications tune codon-anticodon decoding. Wobble-position modifications on tRNAs, including mcm5s2U, m7G, and f5C, are critical determinants of decoding efficiency and accuracy. By modulating the geometry and thermodynamics of the codon-anticodon helix, these marks enable cancer cells to establish codon-biased translation programmes that preferentially synthesize oncogenic proteins [21,62].

Fourth, modifications protect RNA from degradation or quality-control surveillance. m6 Am confers resistance to mRNA decapping [24], pseudouridine at stop codons can suppress translation termination and thereby circumvent nonsense-mediated mRNA decay (NMD) [31] and Nm broadly increases resistance to endonucleolytic cleavage. In each case, the modification extends the functional lifetime or coding potential of the transcript, with direct consequences for protein output.

These four principles are not mutually exclusive: a single modification can simultaneously alter RNA structure, modulate reader protein binding, and influence decoding kinetics. This multifunctionality, combined with the fact that individual transcripts typically carry multiple modification types, generates a combinatorial regulatory space that cancer cells exploit to fine-tune their translational programmes with remarkable precision.

### The cytoplasmic translation cycle: a modification-annotated overview

**Figure 3.**
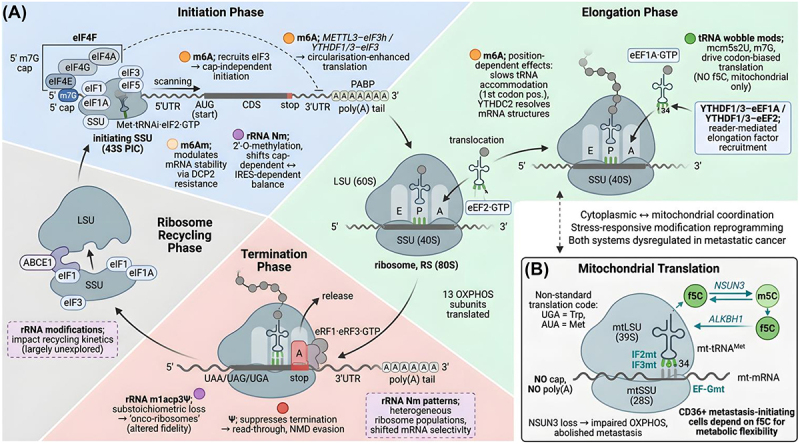
The translation cycle annotated with RNA modification regulatory sites in cancer. (A) The main panel depicts the four phases of cytoplasmic mRNA translation (initiation, elongation, termination, and ribosome recycling) with the major RNA modifications that regulate each stage indicated. During initiation, m6A in 5′ and 3′UTRs, m6 Am at the cap-proximal position, and rRNA 2′-O-methylation (Nm) modulate ribosome recruitment, mRNA circularisation, and the balance between cap-dependent and IRES-dependent translation. During elongation, m6A in coding sequences, wobble-position tRNA modifications (mcm5s2U, m7G, f5C), and reader-mediated elongation factor recruitment (YTHDF1-eEF1A, YTHDF1-eEF2) influence ribosome transit rates and codon-biased translation. At termination, pseudouridine (ψ) at stop codons suppresses release-factor recognition and enables read-through, while substoichiometric rRNA modifications (m1acp3Ψ, Nm) generate functionally heterogeneous ribosome populations (“onco-ribosomes”) with altered translational fidelity and mRNA selectivity. (B) An inset panel shows the mitochondrial translation system, highlighting the dependence of mitoribosome function on the f5C wobble modification of mt-tRnamet (via the NSUN3-ALKBH1 pathway) and the non-standard codon usage (UGA = trp, AUA = Met) that distinguish mitochondrial from cytoplasmic decoding. Arrows connecting the two systems indicate the coordination between cytoplasmic and mitochondrial translation that sustains cancer cell bioenergetics and metastatic competence.

Translation proceeds through four operationally distinct phases, initiation, elongation, termination, and ribosome recycling, each of which is subject to regulation by RNA modifications (Figure 3).

Initiation begins with recognition of the mRNA 5′ cap by the eIF4F complex (comprising eIF4E, the scaffolding protein eIF4G, and the RNA helicase eIF4A), followed by recruitment of the SSU (ribosomal small subunit) initiation complex (consisting of the SSU [40S ribosomal subunit], the ternary complex (Met-tRNA_i_^Met^·eIF2·GTP), and accessory factors eIF1, eIF1A, eIF3, and eIF5) which then scans the 5′UTR in an ATP-dependent manner until the start codon is recognized [67,68]. Interaction of eIF4G with poly(A)-binding protein (PABP) can promote mRNA circularization, potentially enhancing re-initiation efficiency [69]. RNA modifications intervene at multiple points in this process: m6A in 5′UTRs recruits eIF3 for cap-independent initiation; m6A in 3′UTRs promotes circularization-dependent translational enhancement *via* METTL3-eIF3h or YTHDF1-eIF3 interactions; m6 Am at the first transcribed nucleotide modulates cap stability; and altered 2′-O-methylation patterns on rRNA shift the balance between cap-dependent and IRES-dependent initiation. These mechanisms are examined in detail in ‘Initiation Control by RNA Modifications’.

During elongation, the ribosome ratchets along the coding sequence, with aminoacyl-tRNA delivery mediated by eEF1A·GTP and translocation driven by eEF2·GTP [70]. Here, modifications in both mRNA and tRNA exert position-dependent effects. m6A within coding sequences can slow tRNA accommodation at the first codon position while paradoxically enhancing elongation through YTHDC2-mediated resolution of mRNA secondary structures. Wobble-position modifications on tRNAs (mcm5s2U, m7G, f5C) modulate codon-biased translation, enabling cancer cells to upregulate proteins enriched in specific codon families. YTHDF1 and YTHDF3 recruit elongation factors (eEF1A, eEF2) to m6A-marked coding regions, directly accelerating elongation of oncogenic transcripts. These elongation-specific mechanisms are the focus of ‘Elongation Control by RNA Modifications’.

Termination occurs when a stop codon (UAA, UAG, or UGA) enters the ribosomal A site and is recognized by the release factor complex eRF1·eRF3·GTP, triggering peptide release [67,70]. Pseudouridylation of the first uridine in stop codons can suppress termination by enabling non-canonical base-pairing with near-cognate tRNAs, converting stop signals into sense codons. This read-through mechanism has implications for NMD evasion and the translation of extended protein isoforms. Substoichiometric changes in rRNA modifications, notably loss of m1acp3Ψ, create heterogeneous ribosome populations (‘onco-ribosomes’) with altered translational fidelity and mRNA selectivity. These termination and quality-control mechanisms are discussed in ‘Termination and Quality Control: RNA Modifications at the End of the Line’.

Following termination, ribosome recycling dissociates the ribosomal subunits for re-use, a step that is rate-limiting under the high translational demand characteristic of cancer cells and that involves the concerted action of recycling factors ABCE1 and the initiation factors eIF1, eIF1A, and eIF3 [71]. How rRNA modifications influence recycling kinetics remains largely unexplored, but altered ribosome composition is expected to affect subunit dissociation and re-entry into the initiation pathway.

### Mitochondrial translation: a parallel, modification-dependent system

Cancer cells must coordinate cytoplasmic translation with a mechanistically distinct system operating in mitochondria. The mitoribosome (28S SSU +39S LSU [ribosomal large subunit]) translates 13 essential OXPHOS complex subunits from leaderless, polycistronic mRNAs using a non-standard genetic code in which UGA encodes tryptophan and AUA encodes methionine [72]. Mitochondrial translation relies on its own initiation (IF2mt, IF3mt) and elongation factors (EF-Tumt, EF-Gmt), and critically depends on RNA modifications for basic function. The wobble-position f5C modification on the single mitochondrial tRNAMet, generated by the sequential action of NSUN3 (m5C deposition) and ALKBH1 (oxidation to f5C), is essential for decoding both AUG and AUA codons [38,39]. In cancer, loss of the m5C methyltransferase NSUN3 severely impairs mitochondrial translation, reduces OXPHOS capacity, and abolishes metastatic competence in CD36-dependent tumour-initiating cells, demonstrating that mitochondrial RNA modifications are not merely housekeeping marks but active participants in malignant progression [38,64]. Although less extensively characterized than their cytoplasmic counterparts, mitochondrial modification-translation relationships represent an emerging frontier with therapeutic potential, particularly for targeting the metabolic flexibility that sustains metastasis.

### Scope and organisation of the following sections

With this framework in place, the following three sections dissect modification-dependent translational control at each cytoplasmic stage in detail: initiation (‘Initiation Control by RNA Modifications’), elongation (‘Elongation Control by RNA Modifications’), and termination with ribosome quality control (‘Termination and Quality Control: RNA Modifications at the End of the Line’). For each stage, we examine the molecular mechanisms, their cancer-specific consequences, and current or emerging therapeutic implications. By organizing the discussion around the translation cycle rather than by modification type, we aim to reveal the functional logic by which epitranscriptomic rewiring drives oncogenic protein synthesis.

## Initiation control by RNA modifications

**Figure 4.**
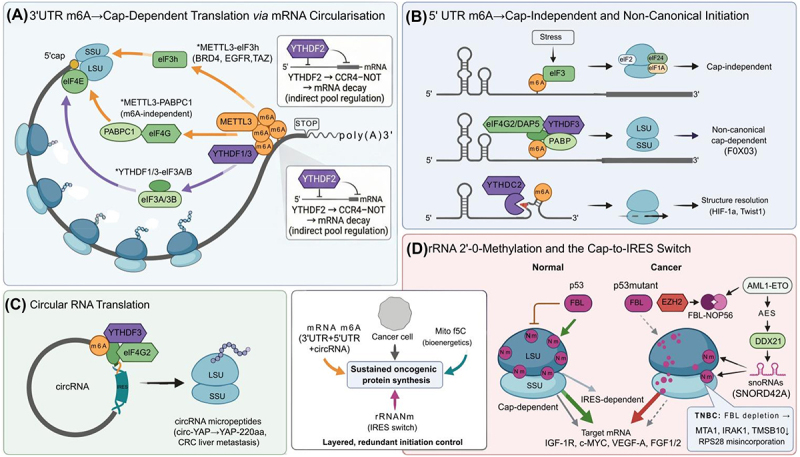
Initiation-stage mechanisms of translational control by RNA modifications in cancer. (A) m6A modifications in 3′UTRs promote cap-dependent translation through mRNA circularisation. Three mechanistically distinct pathways are shown: (i) METTL3 remains associated with its m6A marks and recruits eIf3h, enhancing polysome formation on oncogenes such as *BRD4* and *EGFR*; (ii) METTL3 associates with PABPC1 independently of its methyltransferase activity, strengthening the PABPC1-eIF4G closed-loop interaction; (iii) the m6A readers YTHDF1 and YTHDF3 recruit eIF3A/B to methylated 3′UTRs, with YTHDF3 capable of operating independently in specific tumour contexts. (B) m6A in 5′UTRs enables cap-independent translation initiation. eIF3 directly recognises m6A marks in the 5′UTR and recruits the SSU initiation complex without eIF4E. Stress conditions (hypoxia, drug exposure) redistribute m6A toward 5′UTRs, activating this pathway. eIF4G2 (DAP5) cooperates with YTHDF3 and PABP at 5′UTR m6A sites for a distinct non-canonical cap-dependent route. YTHDC2 unwinds structured 5′UTRs to facilitate ribosome scanning on oncogene mRNAs such as *HIF-1α* and *Twist1*. (C) m6A-dependent translation of circular RNAs. eIF4G2 and YTHDF3 collaborate to initiate translation of circRnas lacking both a 5′ cap and poly(A) tail, generating micropeptides that modulate oncogenic signalling (*e.g*. circ-YAP-derived YAP-220aa in colorectal cancer liver metastasis). (D) rRNA 2′-O-methylation (Nm) directs the cap-to-IRES translational switch. p53 loss leads to FBL overexpression and altered Nm patterns, impairing translational fidelity and enhancing IRES-dependent translation of cancer genes (*IGF-1 R, c-MYC, VEGF-A, FGF1/2*). EZH2 independently promotes this pathway by bridging FBL and NOP56 in the nucleolus. In AML, oncoproteins such as AML1-ETO upregulate C/D box snoRnas (*e.g. SNORD42A*), driving site-specific rRNA methylation that selectively enhances ribosomal protein synthesis. An inset summarises the convergence of these mechanisms: under oncogenic stress, mRNA-level m6A marks, rRNA nm patterns, and mitochondrial f5C modifications jointly ensure sustained translation of survival and proliferation programs.

Translation initiation is the principal rate-limiting step of protein synthesis and is therefore a privileged target for regulation in cancer cells. As outlined in ‘Translation and RNA Modifications: A Mechanistic Framework’, modifications intervene at multiple points during ribosome recruitment, scanning, and start-codon recognition. Here we dissect the molecular mechanisms in detail, beginning with modification-dependent control operating through the mRNA itself (in the 3′UTR, the 5′UTR, and in circular RNAs) before turning to rRNA-level and integrated initiation control (Figure 4).

### m6A in the 3′UTR: promoting cap-dependent translation via mRNA circularization

The enrichment of m6A near stop codons and throughout 3′UTRs positions this modification to influence initiation through the closed-loop model of translation, in which interactions between 3′-end-associated factors and the 5′-cap complex promote ribosome re-initiation *in cis*. Three mechanistically distinct pathways have been defined (Figure 4(a)).

First, METTL3 can function as both writer and reader, remaining associated with the mRNAs it methylates and directly recruiting translation initiation factors. In lung adenocarcinoma, METTL3 binds m6A sites in the 3′UTRs of the oncogenes *BRD4*, *EGFR*, and *TAZ*, and interacts with the eIF3 subunit eIF3h to promote polyribosome formation [44,73]. Tethering experiments confirm that artificial recruitment of METTL3 near stop codons is sufficient to enhance cap-dependent translation, and that the effect depends on the eIF3h interaction rather than on catalytic activity *per se* [73]. Depletion of METTL3 shifts its target mRNAs from heavy polysomal to sub-polysomal fractions, consistent with a defect in initiation rather than elongation [44].

Second, METTL3 can enhance translation independently of its methyltransferase activity. In gastric cancer cells, fewer than 5% of METTL3-bound transcripts carry m6A at the binding site, and both wild-type and catalytically dead METTL3 stimulate luciferase reporter translation [74]. Mechanistically, METTL3 associates with PABPC1, strengthening the PABPC1-eIF4G interaction and thereby reinforcing the closed-loop configuration. This non-canonical pathway broadens the translational impact of METTL3 well beyond m6A-marked transcripts, helping to explain why METTL3 overexpression has such pervasive oncogenic effects across epithelial cancers [74].

Third, the YTH-domain reader proteins YTHDF1 and YTHDF3 mediate m6A-dependent translational enhancement by recruiting eIF3 subunits (eIF3A and eIF3B) to methylated 3′UTRs [41,75]. YTHDF3 was initially characterized as a cooperative partner of YTHDF1, but subsequent work in ovarian and other cancers has shown that YTHDF3 can drive translation independently when YTHDF1 levels are low [75]. This redundancy has clinical implications: therapeutic strategies aimed at a single reader may be compensated by paralogue substitution, arguing for approaches that target the writer (METTL3) or the modification itself. By contrast, YTHDF2 primarily accelerates mRNA decay rather than directly promoting translation, acting through recruitment of the CCR4-NOT deadenylase complex; its role in initiation is therefore indirect, modulating the pool of translatable transcripts rather than ribosome loading *per se* [76].

Collectively, these 3′UTR-centred mechanisms illustrate a general principle: m6A marks deposited co-transcriptionally in the nucleus are ‘read’ in the cytoplasm to enhance initiation of specific oncogenic mRNAs, providing cancer cells with a rapid, selective translational boost that does not require transcriptional upregulation.

### m6A in the 5′UTR: cap-independent translation Initiation

A conceptually distinct pathway operates through m6A deposited in 5′UTRs. Meyer et al. demonstrated that eIF3, conventionally regarded as a component of the cap-dependent machinery, can function as a direct m6A reader, binding methylated 5′UTRs to recruit the 43S complex independently of the cap-binding protein eIF4E [77]. Approximately 35% of eIF3 cross-linking sites coincide with m6A peaks, and the pathway is activated by cellular stress. Heat shock, for example, triggers redistribution of m6A towards 5′UTRs and enables cap-independent translation of *Hsp70* mRNA [77].

This stress-responsive switch has obvious relevance to cancer, where microenvironmental stresses routinely suppress cap-dependent translation *via* mTORC1/4E-BP-mediated sequestration of eIF4E. In drug-resistant melanoma cells, m6A deposition in 5′UTRs enables selective translation of resistance-associated mRNAs despite global translational suppression, demonstrating that the eIF3-m6A axis provides an escape route from cap-dependent translation shutdown [78].

A further m6A-dependent route to non-canonical initiation operates through eIF4G2 (DAP5), which is mechanistically distinct from canonical eIF4E-mediated initiation. eIF4G2 lacks the eIF4E-binding domain present in its paralogue eIF4G1 (Figure 4(b)), yet can support translation through synergistic interactions with YTHDF3 and PABP at m6A sites in the 5′UTR [79]. This pathway is particularly important in tumour microenvironments where mTORC1 signalling is compromised, providing a compensatory mechanism for maintaining synthesis of stress-survival proteins such as FOXO3 [79].

A further mechanism involves resolution of inhibitory RNA secondary structures in 5′UTRs. YTHDC2, a DEAH-box RNA helicase that also binds m6A, unwinds structured 5′UTRs to facilitate ribosome scanning. In colorectal cancer, YTHDC2 enhances translation of *HIF-1α* and *Twist1* under hypoxia, promoting epithelial-mesenchymal transition and metastasis [80,81]. Because structured 5′UTRs are a common feature of oncogene mRNAs, this helicase-coupled reading of m6A represents a broadly relevant initiation-promoting mechanism.

### Circular RNA translation

Circular RNAs (circRNAs) lack both a 5′ cap and a 3′ poly(A) tail, yet a growing subset has been shown to undergo translation in an m6A-dependent manner (Figure 4(c)). Yang et al. demonstrated that eIF4G2 and YTHDF3 collaborate to drive translation of both synthetic and endogenous circRNAs, including circZNF609 and circE7 [82]. In some contexts this process requires eIF3A, whereas in others it proceeds *via* an eIF3A-independent route, suggesting the existence of multiple cap-independent pathways for circRNA translation [82]. The functional significance for cancer is becoming apparent: circRNA-encoded micropeptides can modulate oncogenic signalling, immune evasion, and metabolic reprogramming, adding a previously unrecognized layer to the translational output of the cancer cell [83].

### Mitochondrial translation initiation: cancer-specific implications

As introduced in ‘Translation and RNA Modifications: A Mechanistic Framework’, mitochondrial translation relies on a mechanistically distinct initiation system. The key cancer-relevant point for initiation control concerns the wobble-position f5C modification on the single mitochondrial tRNAMet, generated by the NSUN3-ALKBH1 pathway, which enables decoding of both AUG and AUA codons [38,39]. Delaunay et al. demonstrated that NSUN3 loss severely impairs mitochondrial translation, reduces oxidative phosphorylation capacity, and abolishes metastatic competence specifically in CD36-dependent tumour-initiating cells, a metabolically defined subpopulation critical for cancer dissemination [64]. Mitochondrial initiation control therefore represents a mechanistically distinct but functionally convergent axis through which RNA modifications shape cancer cell fitness, and one that may be particularly relevant for anti-metastatic therapeutic strategies.

### rRNA 2′-O-methylation and the shift to IRES-dependent translation

While the mechanisms described above operate primarily through mRNA-level marks, ribosomal RNA modifications exert an equally consequential and mechanistically distinct influence on initiation. The > 100 sites of 2′-O-methylation (Nm) in human rRNA are installed by the methyltransferase fibrillarin (FBL), guided to specific positions by C/D box small nucleolar RNAs (snoRNAs) [26]. Because Nm stabilizes rRNA helices and modulates the geometry of functional centres, altered methylation patterns do not simply degrade ribosome performance; they redirect the mode of translation initiation (Figure 4(d)).

The seminal work by Marcel et al. established a direct link between p53 loss, FBL overexpression, and translational reprogramming in cancer [25]. In p53-deficient cells, elevated FBL levels alter the rRNA Nm landscape, impair translational fidelity (increased stop-codon read-through and amino acid misincorporation), and shift the balance from cap-dependent towards IRES-dependent initiation. This shift selectively enhances translation of cancer-related IRES-containing mRNAs, including those encoding *IGF-1 R*, *c-MYC*, *VEGF-A*, and *FGF1/2*, drivers of proliferation, angiogenesis, and survival, respectively [25]. Erales et al. subsequently demonstrated that not all Nm sites are equally sensitive to FBL perturbation: a subset of fractionally methylated positions exhibits the greatest plasticity, suggesting that these sites function as tunable regulatory switches rather than constitutive structural marks [26].

The regulatory circuitry governing FBL has expanded considerably since these initial discoveries. Yi et al. revealed that EZH2 (the catalytic subunit of PRC2, long studied as a transcriptional repressor) exerts a PRC2-independent function in the nucleolus, where it bridges FBL and NOP56 to facilitate snoRNP assembly and rRNA 2′-O-methylation [84]. EZH2 deficiency in prostate cancer cells impairs global translation and specifically reduces IRES-dependent initiation, establishing EZH2 as an upstream organizer of the ribosome modification programme. This finding is therapeutically noteworthy, because EZH2 inhibitors (*e.g*. tazemetostat, FDA-approved since 2020) could in principle suppress oncogenic IRES-dependent translation as a non-canonical component of their anti-tumour activity.

At the level of individual snoRNAs, site-specific effects on translation are becoming clearer. In AML, SNORD42A (which directs 2′-O-methylation at 18S rRNA position U116) is overexpressed relative to normal haematopoietic cells, and its deletion selectively reduces translation of ribosomal protein mRNAs [85]. The AML1-ETO oncoprotein drives snoRNA expression through the AES-DDX21 axis, directly coupling a leukaemogenic transcription factor to ribosomal modification and translational output [86]. In solid tumours, very recent work by Groza et al. demonstrated that FBL knockdown in triple-negative breast cancer (TNBC) reduces translation efficiency of the oncogenes *MTA1*, *IRAK1*, and *TMSB10*, and additionally alters 18S rRNA structure in a manner that decreases RPS28 incorporation into ribosomes, providing direct evidence that Nm-dependent ribosome heterogeneity shapes the oncogenic proteome [87].

The clinical significance of these findings is increasingly evident. FBL overexpression correlates with poor prognosis in breast, prostate, hepatocellular, and cervical cancers [25,88]. Strategies to target FBL, whether directly or through upstream regulators such as p53 reactivation or EZH2 inhibition, represent a promising axis for disrupting the cap-to-IRES translational switch that sustains many aggressive cancers.

### Integration: converging initiation mechanisms in cancer

The initiation-stage mechanisms detailed above do not operate in isolation. In a single cancer cell, m6A in 3′UTRs can enhance closed-loop-dependent re-initiation of oncogenic mRNAs, while simultaneously, m6A in 5′UTRs provides cap-independent escape routes when mTORC1 signalling is suppressed by microenvironmental stress. Altered rRNA Nm patterns shift the ribosome population towards IRES-competent configurations, further favouring translation of survival factors under conditions that impair canonical cap-dependent scanning. Mitochondrial initiation modifications sustain the bioenergetic capacity required for all of these cytoplasmic programmes. The result is a layered, redundant system in which multiple modification types converge to ensure that cancer cells maintain oncogenic protein synthesis under virtually any condition, a property that simultaneously represents a therapeutic challenge (because single-target inhibition may be compensated) and a therapeutic opportunity (because combination approaches targeting multiple modification axes can achieve synergistic effects, as discussed in ‘Therapeutic Targeting of RNA Modifications in Cancer Translation’).

Take-home: at initiation, RNA modifications act as selectivity filters rather than simple on/off switches. For example, 3′UTR m6A and rRNA 2′-O-methylation bias which mRNAs are efficiently recruited, while 5′UTR m6A and altered rRNA methylation provide cap-independent escape routes that allow cancer cells to sustain oncogenic protein synthesis when canonical, cap-dependent initiation is suppressed.

## Elongation control by RNA modifications

**Figure 5.**
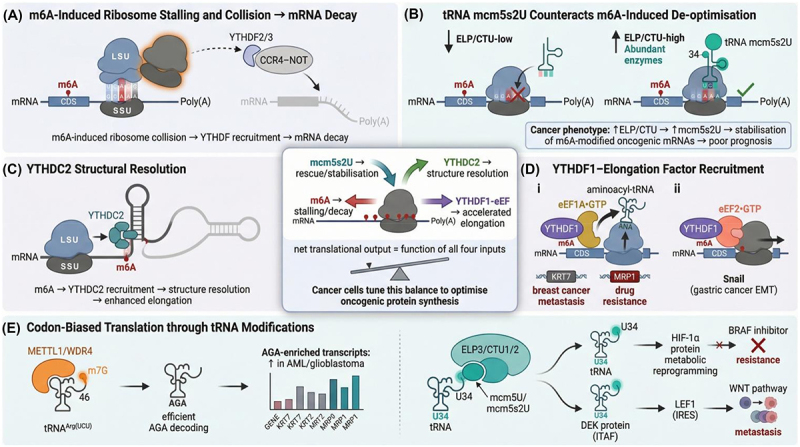
Elongation-stage mechanisms of translational control by RNA modifications in cancer. (A) m6A in the coding sequence impedes ribosome decoding by favouring a π-stacked codon conformation that delays tRNA accommodation. At high m6A stoichiometry, stalling is sufficiently pronounced to induce ribosome collisions, which adopt a unique conformation and recruit YTHDF reader proteins to initiate mRNA degradation through CCR4-NOT-dependent deadenylation. (B) The tRNA anticodon modification mcm5s2U, installed by the ELP/CTU1/2 pathway, counteracts m6A-induced codon de-optimisation: mcm5s2U-modified tRnas decode m6A-containing codons more efficiently, alleviating stalling and stabilising the transcript. In cancer, upregulation of ELP/CTU enzymes shifts the balance toward transcript stabilisation, favouring oncogenic mRNA expression. (C) YTHDC2 paradoxically enhances elongation of m6A-marked transcripts by unwinding inhibitory mRNA secondary structures ahead of the ribosome. (D) YTHDF1 directly recruits elongation factors (eEF1A for aminoacyl-tRNA delivery; eEF2 for translocation) to CDS m6A sites, accelerating elongation of specific oncogenic mRNAs such as *KRT7* (breast cancer metastasis), *Snail* (gastric cancer EMT), and *MRP1* (drug resistance). (E) Codon-biased translation through tRNA modifications. METTL1-dependent m7G46 stabilises tRNA^Arg^(UCU), enhancing translation of AGA-enriched cell cycle mRNAs. ELP3/CTU-dependent wobble modifications (mcm5U/mcm5s2U) selectively promote translation of HIF-1α and glycolytic enzymes in drug-resistant melanoma, and of the ITAF DEK in breast cancer metastasis. An inset illustrates the convergence of these mechanisms: the net translational output of any CDS-modified transcript is determined by the balance between m6A-induced stalling, tRNA modification-dependent rescue, reader-mediated elongation factor recruitment, and structural resolution.

Translation elongation provides cancer cells with a level of regulatory nuance that is distinct from the binary decisions governing initiation. Rather than switching translation on or off, modifications within coding sequences (CDS) and on tRNAs modulate the rate and fidelity of ribosome progression, enabling selective enhancement or repression of specific transcripts while maintaining overall translational capacity (Figure 5). Recent work has revealed that this control extends beyond simple kinetic effects: CDS modifications couple elongation dynamics to mRNA stability, creating an integrated system through which cancer cells coordinate protein output with transcript turnover [63,89].

### m6A in coding sequences: ribosome stalling, collisions, and coupled mRNA decay

The functional consequences of m6A within coding sequences are position-dependent and context-specific. Structural and kinetic studies in bacterial systems demonstrate that m6A at the first position of a codon delays tRNA accommodation by favouring a π-stacked codon conformation that competes with canonical Watson-Crick pairing during A-site decoding [90,91]. In eukaryotic translation systems, m6A at the first codon position substantially impedes elongation through steric hindrance that disrupts the codon-anticodon helix, whereas the effect is markedly reduced when the modification occupies the second or third position [92].

Two landmark 2025 studies have fundamentally reframed how CDS m6A influences transcript fate. Murakami et al. demonstrated that m6A is a potent inducer of ribosome stalling (Figure 5(a)) in mammalian cells, with the degree of stalling, which varies by codon identity, correlating directly with the extent of m6A-mediated mRNA degradation [89]. Stalls induced by m6A are sufficiently pronounced to cause ribosome collisions that adopt a unique conformation distinct from collisions arising in other contexts, and prolonging these collisions enhances degradation. Critically, stalling and collision at m6A sites is followed by recruitment of YTHDF reader proteins, suggesting that altered ribosome dynamics, and not direct reader binding alone, initiate the degradation programme [89].

In a complementary study, Linder et al. showed that m6A-containing codons behave as ‘non-optimal’ codons during elongation, reducing codon optimality and marking transcripts for increased decay through a translation-dependent mechanism [63]. This pathway is modulated by the tRNA anticodon modification mcm5s2U (5-methoxycarbonylmethyl-2-thiouridine), which alleviates the decrease in codon optimality imposed by m6A. In cells depleted of mcm5s2U (through *ELP1* or *CTU2* knockout), ribosome pausing at m6A-modified codons is intensified and m6A-modified mRNAs decay more rapidly. The coordinated regulation extends to entire mRNA regulons, including those encoding oncogenic signalling pathways: in cancer, a shift towards higher mcm5s2U levels is associated with more aggressive tumours and poor prognosis, consistent with a model in which tRNA modifications buffer against m6A-imposed translational pausing to stabilize oncogenic transcripts [63].

These findings establish a previously unrecognized layer of the genetic code, a ‘hidden’ interplay between mRNA and tRNA epitranscriptomes that directly links elongation kinetics to mRNA decay and is co-opted by cancer cells to fine-tune oncogenic gene expression.

Paradoxically, m6A in coding sequences can also enhance translation under certain conditions. Mao et al. demonstrated that m6A promotes elongation by recruiting the RNA helicase YTHDC2, which unwinds inhibitory mRNA secondary structures that would otherwise pause ribosome progression [81]. In cancer cells with elevated mRNA secondary structure content, this m6A-YTHDC2 axis ensures efficient translation of structurally complex oncogenic mRNAs. The opposing effects, m6A-induced stalling at the level of codon decoding *versus* m6A-mediated structural resolution at the level of mRNA architecture, are not contradictory but reflect the context-dependence of modification function: the net outcome depends on local sequence, structure, tRNA availability, and reader protein concentrations.

### YTHDF-elongation factor interactions: direct coupling of m6A to the elongation machinery

Beyond the ribosome collision pathway described above, m6A readers of the YTHDF family directly recruit elongation factors to CDS m6A sites, providing a distinct mechanism for enhancing elongation of specific oncogenic transcripts. In breast cancer lung metastasis, YTHDF1 binds m6A sites within the keratin 7 (*KRT7*) coding region (Figure 5(d)) and recruits eEF1A, accelerating aminoacyl-tRNA delivery and driving enhanced *KRT7* translation that promotes metastatic colonization [93]. Gastric cancer cells exploit an analogous YTHDF1-eEF2 interaction to promote translation of the EMT transcription factor Snail: m6A marks in the *Snail* CDS, rather than its 3′UTR, are the functional determinants, with eEF2-mediated ribosome translocation specifically accelerated at these sites [94].

These reader-elongation factor interactions also contribute to drug resistance. In imatinib-resistant gastrointestinal stromal tumour (GIST) cells, elevated METTL3 deposits m6A on *MRP1* (*ABCC1*) mRNA, which is then recognized by YTHDF1; the resulting YTHDF1-eEF1A complex enhances *MRP1* translation, increasing drug efflux and conferring resistance [95]. The specificity of these interactions is supported by loss-of-function data: FTO overexpression, which removes m6A from target transcripts, specifically reduces YTHDF1-eEF1A binding and diminishes translation of cognate mRNAs without globally perturbing elongation [93].

### tRNA modifications: codon-biased translation in cancer

tRNA modifications represent the most direct mechanism through which the epitranscriptome reprograms elongation, because they alter the efficiency and accuracy with which ribosomes decode individual codons. Cancer cells exploit this system to selectively enhance translation of mRNAs whose codon usage matches the modification-expanded decoding capacity of their tRNA pools, a phenomenon termed codon-biased translation [96].

The best-characterized example involves the Elongator complex (ELP1-6) and its downstream effectors CTU1/CTU2, which catalyse wobble uridine modifications (mcm5U and mcm5s2U) at position 34 of specific tRNAs. In BRAFV600E-mutant melanoma, *ELP3* overexpression drives a codon-biased translational programme that enhances synthesis of HIF-1α and glycolytic enzymes, sustaining a metabolic state that confers resistance to BRAF/MEK inhibitors such as vemurafenib and trametinib [62]. The mechanism is elegantly specific: HIF1A mRNA is enriched in codons requiring U34 modification for efficient decoding, so that ELP3-CTU1/2-mediated modifications act as a bottleneck whose relief preferentially enhances HIF-1α translation [62]. In breast cancer, the same wobble modification system promotes translation of the nuclear oncoprotein DEK through codon-biased elongation; DEK in turn acts as an IRES trans-acting factor (ITAF) that drives IRES-dependent translation of the WNT effector *LEF1*, coupling elongation-level control to initiation-level reprogramming in a cascade that supports metastatic progression [97].

The relevance of the m6A-mcm5s2U interplay described in ‘m6A in Coding Sequences’ extends this model further. Because mcm5s2U counteracts m6A-induced codon de-optimization, cancer cells that upregulate ELP/CTU-dependent modifications effectively stabilize m6A-modified oncogenic transcripts that would otherwise be degraded through ribosome-collision-triggered decay [63]. Pan-cancer analyses corroborate this: tumours with high mcm5s2U relative to m6A levels display worse prognosis, consistent with a protective effect of tRNA modifications on the oncogenic transcriptome [63].

METTL1-dependent m7G modification at position 46 of tRNAs provides a second, mechanistically distinct axis of codon-biased elongation control. m7G46 stabilizes tRNA tertiary structure, increasing the functional pool of specific tRNAs [notably tRNAArg(UCU)] whose cognate AGA codons are over-represented in mRNAs encoding cell cycle regulators [21]. METTL1 amplification or overexpression is recurrent across AML, glioblastoma, liposarcoma, and other tumour types, and its depletion selectively impairs translation of AGA-enriched transcripts while leaving global translation largely intact [21]. Recent pan-cancer analyses of 73 tRNA modification-related genes confirm that tRNA modifier expression is broadly associated with genomic instability, proliferative programmes, and poor prognosis – particularly in breast cancer, where the correlation is strongest in HER2-positive and Luminal A subtypes [98].

A further specialized role is played by ALKBH8, which catalyses wobble modifications on selenocysteine tRNAs, enhancing the synthesis of selenoproteins that confer oxidative stress resistance. In bladder cancer, ALKBH8 overexpression correlates with aggressive disease, consistent with enhanced selenoprotein-mediated survival under the oxidative microenvironment of rapidly growing tumours [99].

### Pseudouridine and elongation fidelity

Pseudouridine (Ψ) introduces distinct effects on elongation fidelity that are separate from its well-characterized role in stop-codon readthrough (discussed in ‘Termination and Quality Control: RNA Modifications at the End of the Line’). *In vitro* reconstitution experiments by Eyler et al. demonstrated that Ψ at the first and third positions of phenylalanine codons significantly increases valine misincorporation, whereas Ψ at the second position has minimal effect, a pattern that parallels the position-dependent effects of m6A but through a different structural mechanism [100]. Whether cancer cells exploit Ψ-mediated miscoding to generate functionally altered protein isoforms remains an open question, though the potential for neoantigen generation through modification-induced amino acid substitution is an intriguing possibility with implications for immunotherapy responsiveness.

### Integration: converging elongation mechanisms in cancer

The elongation-control mechanisms described above form an interconnected regulatory network rather than a set of independent pathways. On any given mRNA, CDS m6A can simultaneously slow ribosome progression (promoting decay), recruit YTHDC2 (relieving structural barriers), and attract YTHDF1-eEF1A/eEF2 complexes (locally accelerating elongation). tRNA modifications (particularly mcm5s2U) modulate the amplitude of m6A-induced pausing and thereby the stability of the transcript. The net translational output is determined by the balance of these opposing forces, providing cancer cells with a sophisticated rheostat for adjusting protein synthesis rates transcript by transcript. This integrated view implies that therapeutic strategies targeting elongation control may need to address multiple modification axes simultaneously, a point developed further in ‘Therapeutic Targeting of RNA Modifications in Cancer Translation’.

Take-home: at elongation, RNA modifications do not switch synthesis on or off but tune the rate and fidelity of ribosome progression transcript by transcript. The output of any coding sequence-modified mRNA reflects a balance between m6A-induced stalling and decay, tRNA modification-dependent rescue (notably mcm5s2U), reader-mediated elongation factor recruitment, and structural resolution, giving cancer cells a means of selectively favouring oncogenic transcripts.

## Termination and quality control: RNA modifications at the End of the Line

**Figure 6.**
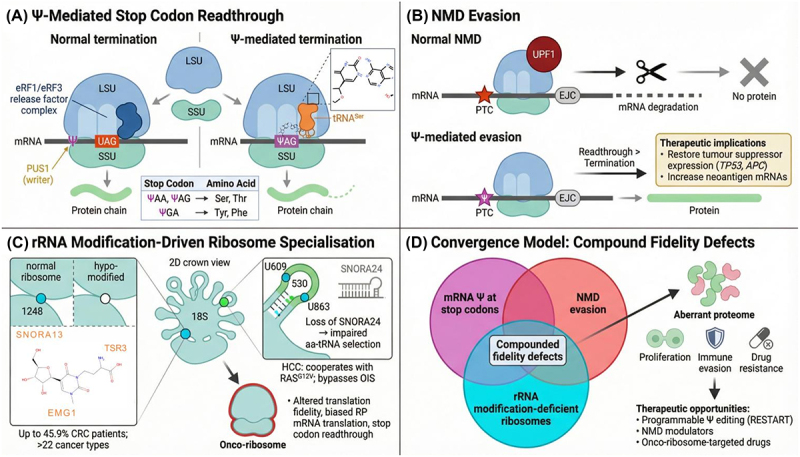
Termination-stage and quality control mechanisms of translational regulation by RNA modifications in cancer. (A) Pseudouridine-mediated stop codon readthrough. When the first uridine of a stop codon (UAA, UAG, or UGA) is pseudouridylated by PUS enzymes (*e.g*. PUS1), the resulting ΨAA/ΨAG/ΨGA codon is recognised by near-cognate tRnas through non-canonical base pairing (Ψ-A Watson-Crick/Hoogsteen pair at position 1, purine-purine pairs at positions 2 and 3). This converts the stop codon to a sense codon, directing incorporation of serine/threonine (ΨAA, ΨAG) or tyrosine/phenylalanine (ΨGA) and producing C-terminally extended readthrough products. (B) Implications for NMD evasion. Top: a transcript bearing a premature termination codon (PTC) is normally recognised by NMD surveillance machinery (UPF1/SMG1), leading to mRNA degradation and loss of protein product. Bottom: pseudouridylation of the PTC enables ribosome readthrough, producing full-length protein and effectively bypassing NMD-mediated transcript elimination. In cancer, this mechanism may restore expression of mutated tumour suppressors or, conversely, allow translation of oncogenic truncated proteins that would otherwise be degraded. (C) rRNA modification-driven ribosome specialisation. Left: loss of the hypermodified base m1acp3Ψ at nucleotide 1248 of 18S rRNA (peptidyl decoding site), present in up to 45.9% of colorectal carcinoma patients and across > 22 cancer types, generates “hypo-m1acp3Ψ” onco-ribosomes with a translational signature enriched for ribosomal protein mRNAs. Right: reduced SNORA24 expression in hepatocellular carcinoma leads to loss of pseudouridylation at U609 (530 loop, shoulder domain) and U863 of 18S rRNA, producing ribosomes with impaired aminoacyl-tRNA selection fidelity and increased stop codon readthrough; this translational infidelity bypasses RAS-induced senescence. (D) Convergence model. Cancer cells may simultaneously harbour mRNA-level Ψ at stop codons (panel a), NMD evasion (panel b), and rRNA modification-deficient ribosomes (panel c), creating compounded fidelity defects that erode translational quality control and generate a permissive environment for the accumulation of aberrant oncogenic proteins.

Translation termination and ribosome-associated quality control represent uniquely consequential nodes for RNA modification-mediated regulation in cancer. Whereas initiation and elongation modifications principally adjust the rate of protein synthesis, modifications that alter termination can change what proteins are made, converting premature or even normal stop codons into sense codons, generating C-terminally extended proteoforms, and enabling transcripts to escape nonsense-mediated mRNA decay (NMD). Cancer cells, with their characteristically high mutational burden, stand to gain disproportionately from such mechanisms. This section examines the molecular basis and cancer relevance of modification-dependent termination control, NMD evasion, and ribosome specialization through rRNA modification (Figure 6).

### Pseudouridine-mediated stop codon readthrough

The discovery that pseudouridylation of stop codons converts them into sense codons provided the first direct evidence that an RNA modification could fundamentally alter the genetic code at the level of translation termination [31]. When the first uridine of any stop codon (UAA, UAG, UGA) is isomerized to pseudouridine (Figure 6a), the resulting ΨAA, ΨAG, or ΨGA triplet is decoded by near-cognate tRNAs: ΨAA and ΨAG direct incorporation of serine and threonine, while ΨGA encodes tyrosine and phenylalanine [31]. Structural analyses of bacterial 30S ribosomal subunits bound to ΨAG-containing codons reveal the molecular basis for this recoding: pseudouridine promotes formation of an unprecedented Ψ-A base pair at the first position *via* Watson-Crick/Hoogsteen geometry, accompanied by purine-purine pairs at the second and third positions [101].

Crucially, Ψ-mediated readthrough is sequence context-independent: when premature termination codons (PTCs) are introduced at different positions within an mRNA and pseudouridylated in yeast, comparable levels of readthrough are observed regardless of the surrounding sequence, and mutational analysis of flanking nucleotides confirms minimal influence on suppression efficiency [102]. This contrasts with conventional stop codon readthrough events, which are strongly context-dependent, and implies that Ψ-mediated recoding is governed primarily by the chemical properties of the modified nucleotide itself.

The transition from engineered to endogenous systems was achieved by quantitative transcriptome-wide mapping using BID-seq (bisulphite-induced deletion sequencing), which confirmed that pseudouridine occurs naturally within stop codons of mammalian mRNAs [60]. In mouse tissues, BID-seq detected Ψ-modified stop codons with stoichiometries reaching ~42% (*e.g*. in *Selenof* mRNA in kidney), corresponding to estimated readthrough levels of up to 35%. Importantly, the degree of readthrough varied by tissue, pointing to tissue-specific regulatory mechanisms that remain to be fully characterized [60]. Among the pseudouridine synthases responsible, PUS1 has emerged as a key writer for stop codon Ψ: depletion of PUS1 reduces both Ψ stoichiometry and translational readthrough, whereas targeted recruitment of PUS1 enhances it [60].

The therapeutic and biotechnological implications of Ψ-mediated readthrough have advanced rapidly. Luo et al. developed RESTART v3, a programmable pseudouridylation system that uses engineered guide RNAs and near-cognate tRNAs to achieve efficient and precise readthrough of disease-associated PTCs, substantially improving on earlier approaches [103]. Most recently, the discovery that pseudouridylation of both the stop codon and the matching tRNA anticodon (a Ψ-Ψ pair) further improves readthrough specificity has opened a new avenue for codon expansion strategies [104]. These advances are directly relevant to cancers driven by nonsense mutations in tumour suppressor genes such as TP53 and APC, where therapeutic restoration of full-length protein through targeted pseudouridylation represents a conceptually distinct approach to conventional gene therapy.

### N1-methylpseudouridine and translation fidelity

N1-methylpseudouridine (m1Ψ), the modification used in COVID-19 mRNA vaccines to reduce innate immune recognition, also influences termination and fidelity, though more subtly than Ψ. Under standard conditions, m1Ψ does not substantially alter the rate constants for cognate tRNA addition or release factor-mediated termination [8]. However, *in vitro* and cellular analyses demonstrate that m1Ψ can modulate translation fidelity in a codon-position and tRNA-dependent manner, with increased amino acid misincorporation at specific positions [7]. While the cancer relevance of m1Ψ remains largely theoretical, the finding that even subtle fidelity changes can generate immunogenic peptides has implications for the design of modified mRNA-based cancer vaccines, where controlled miscoding could expand the neoantigen repertoire presented to the immune system.

### Pseudouridylation and NMD evasion

The intersection of Ψ-mediated stop codon readthrough and NMD has particular significance for cancer biology. NMD normally degrades transcripts that terminate translation prematurely, such as those arising from nonsense mutations, thereby preventing the accumulation of truncated, potentially dominant-negative proteins. In cancer, NMD plays a paradoxical dual role (Figure 6(b)): it functions as a tumour suppressor by eliminating transcripts encoding truncated oncoproteins, yet cancer cells simultaneously exploit NMD to degrade transcripts from tumour suppressor genes that have acquired nonsense mutations, and to eliminate mRNAs encoding immunogenic neoantigens [105]. The selective pressure on the NMD pathway in tumours with high mutational loads as in microsatellite-instable colorectal cancers or UV-associated melanomas creates a context in which modification-dependent NMD evasion could confer significant fitness advantages.

When a PTC is pseudouridylated, the resulting amino acid incorporation converts what would be a termination event into a continuation of elongation, bypassing the requirement for eRF1/eRF3 recognition that normally triggers NMD surveillance. Importantly, in the yeast system, targeted PTC pseudouridylation does not appear to suppress NMD directly at the RNA level; rather, the readthrough occurs at the translational level, with the ribosome simply failing to recognize the modified codon as a stop signal [102]. The net effect is that pseudouridylated transcripts bearing PTCs can produce full-length or near-full-length proteins while the mRNA itself may still be subject to NMD surveillance, the critical variable being the competition between readthrough and termination kinetics at the modified codon.

From a therapeutic perspective, the dual nature of NMD in cancer presents both opportunities and challenges. NMD inhibition has been proposed as a strategy to restore expression of tumour suppressor transcripts bearing PTCs and to increase the abundance of neoantigen-encoding mRNAs, thereby enhancing anti-tumour immune responses [106]. Pharmacological NMD inhibitors targeting UPF1 or SMG1 are under preclinical investigation for this purpose. Targeted pseudouridylation offers an orthogonal approach: rather than globally inhibiting NMD (which would also stabilize many off-target transcripts), programmable Ψ editing could selectively restore specific tumour suppressor proteins at the translational level while leaving NMD function intact for other transcripts. The RESTART platform and its successors provide the molecular tools to test this concept, though delivery to solid tumours remains a significant hurdle [103].

### rRNA modifications and the onco-ribosome

The long-standing assumption that all ribosomes within a cell are structurally and functionally equivalent has been increasingly challenged, although the extent to which rRNA modification heterogeneity translates into *bona fide*, selective ‘specialised ribosome’ function, as opposed to graded quantitative effects on overall ribosome performance, remains debated and is difficult to establish rigorously. Substoichiometric variation in rRNA modifications generates ribosome populations with distinct translational properties, and in cancer, the resulting ‘onco-ribosomes’ preferentially translate mRNA subsets that support malignant programmes [107]. Two landmark studies illustrate how specific rRNA modification losses create cancer-permissive ribosome variants through effects on termination and translational fidelity. We note, however, that while substoichiometric variation in rRNA modifications is well documented, demonstrating that distinct ribosome subpopulations selectively translate defined mRNA subsets *in vivo* remains technically demanding; the term ‘onco-ribosome’ is therefore best understood as a useful operational descriptor rather than a firmly established functional entity.

#### m1acp3Ψ loss at the peptidyl decoding site

Babaian et al. identified a cancer-specific reduction in 1-methyl-3-α-amino-α-carboxyl-propyl pseudouridine (m1acp3Ψ) at nucleotide 1248 of 18S rRNA, a hypermodified base that has been conserved for over one billion years of eukaryotic evolution and resides within the ribosome’s peptidyl decoding site [61]. The ‘hypo-m1acp3Ψ’ phenotype was detected in up to 45.9% of colorectal carcinoma patients and was present across more than 22 cancer types in TCGA data, yet was largely absent from patient-matched normal tissues. The three-step biogenesis of m1acp3Ψ, including SNORA13-guided pseudouridylation, EMG1-mediated N1-methylation, and TSR3-catalysed acp3 transfer, means that disruption at any step can abolish the modification. Functionally, m1acp3Ψ-deficient ribosomes and hypo-m1acp3Ψ patient tumours share a translational signature characterized by elevated ribosomal protein mRNA translation, suggesting that modification loss perturbs decoding in a manner that skews the proteome towards ribosome biogenesis components [61]. These m1acp3Ψ-deficient ribosomes represent a molecularly defined class of onco-ribosome and a potential chemotherapeutic target.

#### SNORA24-guided pseudouridylation and translational fidelity in hepatocellular carcinoma

In a complementary study, McMahon et al. demonstrated that the H/ACA snoRNA *SNORA24*, which guides pseudouridylation at positions U609 and U863 of 18S rRNA, functions as a tumour suppressor downstream of oncogenic RAS [108]. SNORA24 expression is reduced in a subset of human hepatocellular carcinomas (HCC) and its loss cooperates with RASG12V to drive steatohepatitic HCC in mouse models. Ribosomes lacking SNORA24-guided modifications exhibit reduced aminoacyl-tRNA selection fidelity and increased stop codon readthrough consistent with the location of U609 within the functionally critical ‘530 loop’ of the 18S rRNA shoulder domain, which directly contacts the mRNA codon-tRNA anticodon pair during decoding [108]. The resulting translational infidelity bypasses oncogene-induced senescence, providing a mechanism by which a single rRNA modification change can tip the balance from tumour suppression to malignant transformation.

#### Convergence of mRNA and rRNA modification defects

These rRNA modification losses do not operate in isolation from the mRNA-level pseudouridylation described in ‘Pseudouridine-Mediated Stop Codon Readthrough’. A cancer cell that simultaneously harbours hypo-m1acp3Ψ ribosomes, reduced SNORA24-guided Ψ, and elevated mRNA pseudouridylation at stop codons would experience compounded fidelity defects: ribosomes that are inherently less accurate in decoding (rRNA defects) would encounter stop codons that are themselves rendered ambiguous by mRNA modification. Although this convergence has not yet been directly demonstrated experimentally, the co-occurrence of these phenotypes across cancer types, particularly in colorectal carcinoma, where both hypo-m1acp3Ψ and elevated mRNA Ψ have been documented, makes it a compelling area for future investigation. Recent advances in epitranscriptomic rRNA fingerprinting using long-read nanopore sequencing are now enabling simultaneous profiling of multiple rRNA modifications at single-molecule resolution, providing the technical means to test this convergence model across tumour types and at the single-cell level [109].

The 2′-O-methylation landscape of rRNA provides a further axis of ribosome specialization relevant to translational fidelity. As discussed in ‘Initiation Control by RNA Modifications’, altered FBL-dependent 2′-O-methylation patterns shift the balance from cap-dependent to IRES-dependent translation. However, the same methylation changes also reduce overall translational accuracy, increasing amino acid misincorporation and stop codon readthrough [25,26]. The convergence of pseudouridylation defects and 2′-O-methylation alterations in cancer ribosomes suggests that multiple modification axes cooperate to produce the translational infidelity that enables tumour progression – a theme that is explored further in the context of integrated modification networks (Integrated Modification Networks: How Multi-Layered Epitranscriptomic Crosstalk Shapes Malignant Translation).

### Integration: a multi-Layered system for evading translational quality control

The termination and quality control mechanisms described above form a multi-layered system that cancer cells can exploit at several levels simultaneously. At the mRNA level, pseudouridylation of stop codons, whether at PTCs in tumour suppressor genes or at normal stop codons, generates extended proteoforms and enables NMD evasion. At the rRNA level, loss of m1acp3Ψ and SNORA24-guided modifications produces ribosomes with inherently reduced decoding fidelity, compounding the effects of mRNA modification. Together, these mechanisms erode the quality control infrastructure that normally ensures faithful protein synthesis, creating a permissive environment for the accumulation of aberrant proteins that drive malignant phenotypes. The observation that these defects are found across a wide range of cancer types, yet are largely absent from normal tissues, underscores their potential as both diagnostic biomarkers and therapeutic targets. Strategies to exploit these vulnerabilities, from programmable pseudouridylation to restore tumour suppressor expression, to pharmacological NMD modulation to enhance neoantigen presentation, to targeted disruption of onco-ribosome function, represent an emerging frontier in epitranscriptomic cancer therapy.

Take-home: at termination and quality control, RNA modifications can change not only how much protein is made but which protein is made. Stop-codon pseudouridylation and modification-deficient ribosomes erode decoding fidelity and enable readthrough and NMD evasion; however, the *in vivo* magnitude and selectivity of these effects in human tumours remain only partly defined.

## Integrated modification networks: how multi-layered epitranscriptomic crosstalk shapes malignant translation

**Figure 7.**
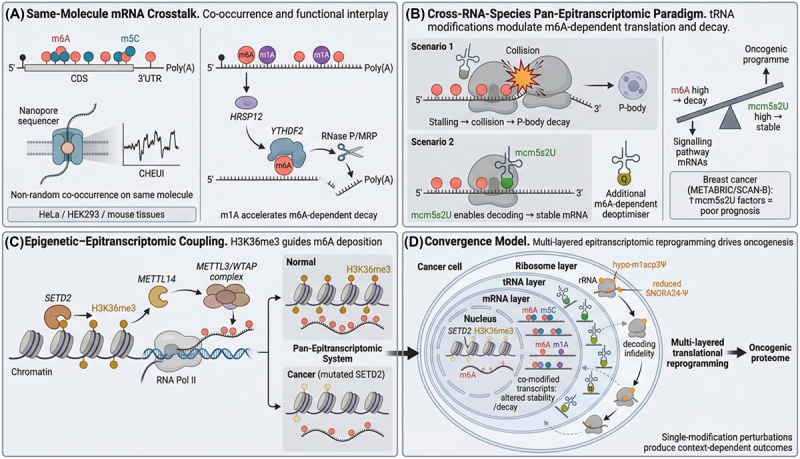
Integrated modification networks in malignant translation: multi-layered epitranscriptomic crosstalk. The figure illustrates three experimentally established levels of RNA modification integration that collectively shape the translational landscape in cancer. (A) Same-molecule mRNA crosstalk. Left: m6A and m5C co-occur non-randomly on individual mRNA molecules, as revealed by single-molecule nanopore sequencing (CHEUI). The co-occurrence patterns differ between normal and cancer cells and may involve coordinated deposition through shared readers or sequential enzymatic dependencies. Right: m1A recruits the adaptor protein HRSP12, which promotes efficient binding of the m6A reader YTHDF2, accelerating endoribonucleolytic cleavage of co-modified transcripts *via* the RNase P/MRP complex. This cooperative m1A-m6A decay mechanism selectively reshapes the translatable mRNA pool. (B) Cross-RNA-species coordination: the pan-epitranscriptomic paradigm. m6A modifications in the mRNA coding sequence render modified codons “non-optimal”, inducing ribosome stalling and collisions that trigger mRNA decay. The tRNA anticodon modification mcm5s2U (installed by the ELP3/CTU1/2 pathway) counteracts this effect by restoring efficient decoding. The balance between mRNA m6A and tRNA mcm5s2U thus acts as a licensing system: oncogenic signalling pathway mRNAs (high m6A) are rapidly turned over, while metabolic housekeeping mRNAs (low m6A) remain stable. In cancer, upregulation of mcm5s2U factors shifts this balance toward stabilisation of signalling-pathway transcripts and correlates with poor prognosis. Queuosine (Q), another tRNA anticodon modification (installed by QTRT1/2), represents an additional deoptimising modification whose effects are m6A-dependent. (C) Epigenetic-epitranscriptomic coupling. The histone mark H3K36me3, deposited co-transcriptionally by the methyltransferase SETD2, is directly recognised by METTL14, recruiting the m6A methyltransferase complex to nascent RNA *via* RNA pol II. SETD2 loss-of-function mutations (frequent in clear cell renal cell carcinoma and other cancers) globally reduce m6A deposition, redistributing the modification across the transcriptome and altering which mRNAs enter the pan-epitranscriptomic decay/stability system described in panel (B). (D) Convergence model. In a cancer cell, the three levels of integration converge: chromatin mutations (SETD2 loss) alter co-transcriptional m6A deposition; same-molecule crosstalk between m6A, m5C, and m1A modulates transcript stability and translation; cross-species interactions between mRNA m6A and tRNA mcm5s2U determine mRNA fate; and rRNA modification defects (hypo-m1acp3Ψ, reduced SNORA24-guided ψ; see “termination and quality control: RNA modifications at the End of the Line”) compound the resulting translational infidelity. Arrows indicate experimentally demonstrated interactions; dashed arrows indicate predicted but not yet directly demonstrated connections. This multi-layered integration explains why single-modification perturbations often produce context-dependent outcomes and underscores the need for network-aware therapeutic strategies.

The preceding sections examined individual RNA modifications at discrete stages of translation. However, cancer cells do not deploy these modifications in isolation. Emerging evidence driven largely by single-molecule and multi-omics technologies indicates that RNA modifications can engage in crosstalk, both within individual transcripts and across RNA species. These interactions may create integrated regulatory networks whose translational outputs cannot easily be predicted from any single modification alone. It is important to emphasize that, while the individual pairwise interactions described below are experimentally supported, their proposed convergence into a single integrated network in any given tumour remains largely a conceptual model that awaits direct, simultaneous measurement. This section examines three experimentally grounded levels of integration: (i) same-molecule crosstalk between mRNA modifications, (ii) cross-species coordination between mRNA and tRNA epitranscriptomes, and (iii) upstream coupling of chromatin state to co-transcriptional modification deposition (Figure 7).

### Same-molecule crosstalk: co-occurring mRNA modifications

The first direct evidence that different modification types co-occur non-randomly on individual mRNA molecules came from the CHEUI (CH3 Estimation Using Ionic current) platform, which applies deep-learning models to nanopore direct RNA sequencing signals to predict m6A and m5C at single-molecule resolution [54]. Analysis of HeLa, HEK293, and mouse tissue transcriptomes revealed that m6A and m5C co-occur on the same transcript molecules more frequently than expected by chance, with co-modified transcripts showing distinct stoichiometric profiles compared with singly modified counterparts [54]. Although the mechanistic consequences of this co-occurrence for translation remain to be fully dissected, the non-random patterns are consistent with coordinated deposition, potentially through shared reader proteins or sequential enzymatic dependencies, rather than independent stochastic modification.

A second, mechanistically characterized axis of mRNA modification crosstalk involves m1A and m6A. Boo et al. demonstrated that m1A accelerates the degradation of m6A-containing transcripts by recruiting the RNA-binding protein HRSP12, which in turn promotes efficient interaction of the m6A reader YTHDF2 with its target, facilitating endoribonucleolytic cleavage *via* the RNase P/MRP complex [59]. Transcriptome-wide analyses confirmed that mRNAs harbouring both m1A and m6A are downregulated in an HRSP12-dependent manner compared with transcripts carrying m6A alone; the same cooperative mechanism extends to endogenous circular RNAs [59]. This finding has direct implications for cancer translation: tumours that upregulate TRMT6-TRMT61A (the m1A writer complex) could accelerate turnover of specific m6A-marked transcripts, selectively reshaping the translatable mRNA pool without altering m6A levels *per se*.

Together, these studies establish a principle with broad translational relevance: the functional impact of any single mRNA modification depends on the modification landscape of the same molecule. For cancer biology, this implies that therapeutic inhibition of one writer (*e.g*. METTL3) may have context-dependent effects dictated by the status of other modifications on the same transcripts, a factor that is rarely considered in current drug development programmes.

### Cross-species coordination: the pan-epitranscriptomic paradigm

A conceptual leap in understanding modification networks came with the demonstration that mRNA and tRNA modifications cooperate to control transcript fate, a mechanism termed ‘pan-epitranscriptomic’ regulation by Linder et al. [63]. Using ribosome profiling combined with genetic and pharmacological perturbation, they showed that m6A-modified codons in the coding sequence are decoded inefficiently by the ribosome, effectively rendering them ‘non-optimal’ and inducing ribosome collisions that couple translation to mRNA decay [63]. Critically, the tRNA anticodon modification 5-methoxycarbonylmethyl-2-thiouridine (mcm5s2U) installed by the ELP3/CTU1/2 pathway counteracts this effect by restoring efficient decoding at m6A-modified codons (Figure 5(b)). The balance between mRNA m6A and tRNA mcm5s2U thus constitutes a ‘licensing system’ that determines whether a given transcript is rapidly degraded or stably translated.

The cancer relevance of this pan-epitranscriptomic axis is striking. Linder et al. found that mRNAs encoding components of oncogenic signalling pathways contain high m6A densities and are rapidly degraded under normal conditions, whereas housekeeping metabolic transcripts carry little m6A and are stable [63]. In breast cancer patient cohorts (METABRIC and SCAN-B), elevated expression of mcm5s2U biogenesis factors was associated with poor prognosis, while high expression of m6A pathway components correlated with better outcomes, which is precisely the relationship predicted by their opposing molecular functions [63]. A pan-epitranscriptomic gene expression signature derived from the relative expression of mcm5s2U *versus* m6A factors provided prognostic stratification independent of conventional markers.

This paradigm extends beyond the m6A-mcm5s2U pair. Linder et al. also identified queuosine (Q), a tRNA modification synthesized by the QTRT1/QTRT2 pathway, as another ‘deoptimising’ anticodon modification whose effects on ribosome occupancy depend on mRNA m6A status [63]. These findings redefine the concept of codon optimality in cancer: it is not codon identity alone, but the interplay between codon-level mRNA modifications and anticodon-level tRNA modifications, that determines translational efficiency and mRNA stability. For the therapeutics discussed in ‘Therapeutic Targeting of RNA Modifications in Cancer Translation’, this implies that drugs targeting mRNA modification writers (*e.g*. METTL3 inhibitors) and those affecting tRNA modification pathways (*e.g*. ELP3 inhibitors) may have synergistic or antagonistic effects depending on the modification state of their shared target transcripts.

### Epigenetic-epitranscriptomic coupling: from chromatin to translation

The modification networks described above operate at the RNA level, but their architecture is shaped by upstream chromatin signals. The landmark demonstration by Huang et al. that histone H3 trimethylation at lysine 36 (H3K36me3), a hallmark of transcriptional elongation, guides co-transcriptional m6A deposition established the first direct mechanistic link between the epigenome and the epitranscriptome [110]. METTL14 directly recognizes and binds H3K36me3, recruiting the m6A methyltransferase complex to RNA polymerase II and enabling site-specific m6A deposition on nascent transcripts; depletion of the H3K36me3 writer SETD2 globally reduces m6A levels across the transcriptome [110].

This coupling has direct cancer implications. SETD2 is among the most frequently mutated chromatin regulators in clear cell renal cell carcinoma (ccRCC), where its loss is associated with metastatic progression and poor survival. Recent work in isogenic ccRCC models confirmed that SETD2 loss redistributes m6A across the transcriptome, with co-regulated targets including genes implicated in immune evasion and chemoresistance [111]. The H3K36me3-m6A axis thus represents a conduit through which cancer-associated chromatin mutations can propagate to the translational level, altering which transcripts are m6A-marked and, consequently, how they are decoded, stabilized, or degraded.

The H3K36me3-METTL14 axis is one instance of a broader, bidirectional dialogue between the epigenome and the m6A epitranscriptome. Co-transcriptional m6A deposition is shaped by additional chromatin features and histone modifications, by the transcription-elongation rate of RNA polymerase II, and by interactions of the methyltransferase complex with chromatin-associated factors; conversely, m6A and its nuclear readers can feed back onto chromatin and transcription, for example through effects on chromatin-associated regulatory RNAs and on the stability of transcripts encoding chromatin modifiers, establishing reciprocal regulatory loops. This crosstalk extends to other modification layers, including interplay between DNA methylation/TET activity and RNA methylation. These bidirectional interactions, recently reviewed in detail [112–114], imply that cancer-associated mutations in chromatin regulators can reshape the epitranscriptome, and hence the translatome, well beyond any single histone-mark/writer pairing.

### Convergence: multi-level integration in cancer

The three layers of modification crosstalk described above – same-molecule mRNA interactions, cross-RNA-species coordination, and epigenetic-epitranscriptomic coupling – do not operate independently. Consider a cancer cell that has lost SETD2 function: the resulting H3K36me3 depletion globally reduces m6A, shifting the mRNA-tRNA modification balance towards mcm5s2U-dominated ‘stable’ decoding and altering the m1A-m6A cooperative decay axis on co-modified transcripts. Simultaneously, if the same cell harbours the rRNA modification defects described in ‘Termination and Quality Control: RNA Modifications at the End of the Line’, such as hypo-m1acp3Ψ ribosomes or reduced SNORA24-guided pseudouridylation, the resulting decoding infidelity compounds the translational consequences of mRNA-level modification changes. The net effect is a multi-layered reprogramming of the translational landscape that no single modification perturbation can fully explain.

If validated, this convergence model (Figure 7) would have important practical implications. First, for biomarkers: multi-modification signatures that integrate mRNA, tRNA, and rRNA modification status (or the relative expression of their biogenesis enzymes) are likely to outperform single-modification markers for prognosis and treatment response prediction, as demonstrated by the pan-epitranscriptomic signature in breast cancer [63]. Second, for therapy: the interconnected nature of modification networks suggests that targeting a single node may trigger compensatory rewiring through alternative modification axes, potentially contributing to treatment resistance. Rational combination strategies that simultaneously perturb multiple network layers (pairing a METTL3 inhibitor with an ELP3 pathway modulator *etc*.) may be required to durably suppress malignant translational programmes. Third, for basic understanding: the field urgently needs experimental platforms that can simultaneously profile multiple modification types on the same molecules, in the same cells, at single-cell resolution. Technologies such as CHEUI-based nanopore analysis and emerging multi-modification long-read sequencing approaches are beginning to provide these capabilities, but their application to primary tumour samples remains in its infancy.

The recognition that RNA modifications function as integrated networks rather than independent switches marks a maturation of the epitranscriptomics field. For cancer biology, this network perspective reveals why single-modification studies have sometimes produced contradictory results; the outcome of any modification depends on its molecular and cellular context, including the status of other modifications on the same transcript, on the tRNAs that decode it, on the ribosomes that translate it, and on the chromatin that directed its initial modification during transcription. Embracing this complexity will be essential for translating epitranscriptomic discoveries into effective cancer therapies.

## Therapeutic targeting of RNA modifications in cancer translation

**Figure 8.**
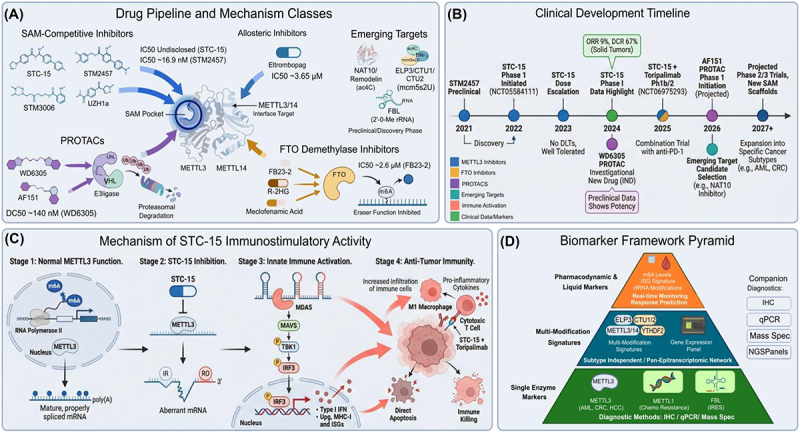
Therapeutic landscape for targeting RNA modifications in cancer translation. (A) Drug pipeline and mechanism classes. The figure maps current and emerging therapeutic agents to their molecular targets within the m6A regulatory system and beyond. METTL3 inhibitors (STC-15, STM2457, STM3006) compete with SAM for the catalytic pocket of the METTL3-METTL14 heterodimer, reducing m6A deposition on mRNA. FTO demethylase inhibitors (FB23-2, R-2HG, meclofenamic acid) block m6A removal, increasing modification levels. METTL3-METTL14 PROTACs (WD6305, AF151) recruit the VHL E3 ligase to induce proteasomal degradation of the entire methyltransferase complex, eliminating both catalytic and non-catalytic (scaffolding, eIF3 recruitment) functions. Emerging targets include NAT10 (ac4C writer), ELP3/CTU1/2 (tRNA wobble modification pathway), and FBL (rRNA 2′-O-methylation). (B) Clinical development timeline. STC-15 Phase 1 dose escalation (NCT05584111, 2022–2024): 42 patients, 60–200 mg TIW, ORR 9%, DCR 67%, no DLTs. STC-15 Phase 1b/2 + toripalimab (NCT06975293, 2025-ongoing): combination with anti-PD-1 in NSCLC, HNSCC, melanoma, and endometrial cancer; up to 188 patients. Preclinical pipeline compounds are shown on a projected timeline toward IND-enabling studies. (C) Mechanism of STC-15 immunostimulatory activity. METTL3 inhibition causes loss of m6A at highly modified long exons, triggering intron retention (IR) and transcriptional run-on (RO) events. The resulting aberrant transcripts form double-stranded RNA (dsRNA) that is sensed by MDA5 in the cytoplasm, activating the type I interferon signalling cascade. This remodels the tumour microenvironment: upregulation of MHC-I and antigen presentation, M1 macrophage polarisation, and secretion of pro-inflammatory cytokines and chemokines - creating a “hot” tumour environment that synergises with PD-1 checkpoint blockade. (D) Biomarker framework. Three tiers of biomarkers for epitranscriptomic therapy: (i) single-enzyme expression (METTL3, FBL, METTL1) as prognostic markers; (ii) multi-modification network signatures, exemplified by the pan-epitranscriptomic score (mcm5s2U biogenesis factors *versus* m6A pathway components) that predicts overall survival in breast cancer; (iii) pharmacodynamic readouts including blood m6A levels and interferon-stimulated gene enrichment for monitoring on-target drug activity.

The mechanistic insights described in the preceding sections, starting from m6A-driven initiation reprogramming all the way to pan-epitranscriptomic mRNA-tRNA coordination, are now converging with drug development. The first RNA modification enzyme inhibitor has completed Phase 1 clinical evaluation and advanced into combination trials, while a rapidly expanding pipeline of small molecules and targeted protein degraders is approaching clinical readiness. This section examines the current therapeutic landscape through the lens of translational control, connecting the biology of modification-dependent translation to actionable intervention strategies (Figure 8).

### STC-15: first-in-class clinical validation of METTL3 inhibition

The clinical development of STC-15 by STORM Therapeutics represents a landmark in epitranscriptomic medicine: it reveals the first pharmacological agent targeting an RNA-modifying enzyme to enter human trials. STC-15 is an orally bioavailable, highly selective inhibitor of METTL3 that competes with S-adenosylmethionine (SAM) for binding in the catalytic pocket of the METTL3-METTL14 heterodimer [115].

The Phase 1 dose-escalation study (NCT05584111) enrolled 42 patients with advanced solid tumours across five dose cohorts (60–200 mg, thrice weekly). Final results, presented at SITC 2024, demonstrated that STC-15 was well tolerated with no dose-limiting toxicities; treatment-emergent adverse events were mild, transient, and manageable with supportive care [9]. Target engagement was robust: rapid and sustained reductions in m6A levels on polyadenylated RNA confirmed on-target pharmacodynamic activity across all dose levels. Tumour regressions were observed at every dose tested, with an overall response rate of 9% and a disease control rate of 67%. Three patients achieved sustained partial responses [9].

Several caveats temper this early-stage optimism. First, the clinical evidence remains preliminary: the STC-15 results derive from a single-arm Phase 1 study (*n* = 42) with an overall response rate of 9%, and such signals will require confirmation in randomized, controlled trials; to date no RNA-modification-targeting agent has demonstrated a survival benefit. Second, RNA-modifying enzymes are not cancer-specific. METTL3, FTO, NAT10 and the ELP/CTU machinery perform essential functions in normal cells, including haematopoietic stem cells, so a therapeutic window must be demonstrated rather than assumed; SAM-competitive METTL3 inhibitors additionally face the general selectivity challenge of competing at a cofactor pocket shared with numerous methyltransferases, and the consequences of chronic m6A reduction in normal tissues are not yet well characterized. FTO inhibitors have a particularly chequered selectivity history, and reports of small-molecule activity against individual modifiers have in some cases been disputed (for example, the ADAR1 inhibitor ZYS-1; see Table 2). Third, the effects of RNA modifications are strongly context-dependent: m6A and its regulators can be oncogenic or tumour-suppressive depending on tumour type, genetic background and the specific transcripts affected, so that even the desired direction of intervention (writer inhibition *versus* eraser inhibition) is itself context-dependent.

**Table 2.** Therapeutic pipeline targeting RNA modification enzymes in cancer.

Compound	Target	Modality	Potency	Development stage	Tumour indication(s)	Key mechanism / notes	Key reference(s)
**STC-15**	METTL3 (SAM-competitive)	Small molecule inhibitor	Undisclosed (clinical grade)	Phase 1b/2 (NCT06975293; first pt dosed May 2025)	NSCLC, HNSCC, melanoma, endometrial (+ toripalimab combination)	m6A loss → IR/RO → dsRNA → MDA5 → type I IFN; ORR 9%, DCR 67% in Phase 1 monotherapy	[9,116] NCT06975293
**STM2457**	METTL3 (SAM-competitive)	Small molecule inhibitor	IC50 = 16.9 nM	Preclinical (tool compound)	AML (preclinical POC)	Selectively reduces m6A on leukaemogenic transcripts; promotes differentiation and apoptosis; extends survival in AML models	[115]
**STM3006**	METTL3 (SAM-competitive, 2nd generation)	Small molecule inhibitor	Improved vs STM2457	Preclinical	Solid tumours, haematological malignancies	Distinct structural features; activity in solid tumour models	[119]
**Eltrombopag**	METTL3-METTL14 interface (allosteric)	Repurposed small molecule	Allosteric inhibitor; IC50 = 3.65 μM	Approved (ITP; repurposing in preclinical)	AML, solid tumours (preclinical)	First allosteric METTL3 inhibitor; targets protein-protein interaction rather than catalytic site	[121]
**UZH1a**	METTL3 (SAM-competitive)	Small molecule inhibitor	IC50 = 280 nM (biochemical); 4.6 μM (cellular)	Preclinical (tool compound)	Various	Structure-based; occupies SAM adenosine pocket; cellular potency limited by SAM competition	[120]
**WD6305**	METTL3-METTL14 complex	PROTAC (VHL-recruiting)	DC50 = 140 nM (METTL3); Dmax ~92%	Preclinical	AML	Near-complete degradation of heterodimeric complex; eliminates both catalytic and scaffolding functions (eIF3 recruitment, mRNA looping)	[125]
**AF151**	METTL3	PROTAC (indole-nicotinamide chemotype)	DC50 = 430 nM	Preclinical	AML	Structurally distinct from UZH2-based degraders; enhanced viability inhibition and m6A reduction	[126]
**FB23-2**	FTO (m6A demethylase)	Small molecule inhibitor	IC50 = 2.6 µM	Preclinical	AML	Increases global m6A → enhanced m6A-dependent mRNA decay; anti-proliferative in AML PDX models	[122]
**R-2HG**	FTO (natural inhibitor)	Oncometabolite (natural)	Competes with α-KG	Endogenous (IDH-mutant cancers)	AML, glioma (IDH-mutant)	Natural FTO inhibitor; intersects cancer metabolism with epitranscriptomic regulation	[123]
**Meclofenamic acid**	FTO	Repurposed NSAID	Selective over ALKBH5	Approved (NSAID; repurposing preclinical)	Various (preclinical)	Selectively inhibits FTO over ALKBH5; proof of repurposing potential	[124]
**Remodelin**	NAT10 (ac4C writer)	Small molecule inhibitor	Limited selectivity	Preclinical	Various solid tumours	Originally developed for laminopathy; anti-cancer activity; NAT10 promotes ac4C-dependent translation of cell-cycle and resistance genes	[127]
**ZYS-1**	ADAR1 (A-to-I editing)	Small molecule inhibitor	Undisclosed	Preclinical	Prostate cancer	Originally reported as first ADAR1 inhibitor with *in vivo* anti-tumour efficacy and synergy with anti-PD-1; however, ADAR1 target specificity has been disputed by independent groups [128]	[129]
**ADAR1i-124**	ADAR1 (p150 + p110)	Small molecule inhibitor	Dose-dependent	Preclinical	Melanoma, various solid tumours	Inhibits both ADAR1 isoforms; activates MDA5 and ZBP1 pathways; synergises with 5-Aza-CdR	[130]
**STC-15 + venetoclax**	METTL3 + BCL2	Combination	Synergistic in PDX	Preclinical	AML	m6A stabilises BCL2 mRNA translation; METTL3 inhibition + BCL2 inhibition = extended survival in AML PDX	[131]
**STC-15 + toripalimab**	METTL3 + PD-1	Combination	Complementary clonal targeting	Phase 1b/2 (NCT06975293)	NSCLC, HNSCC, melanoma, endometrial	METTL3 inhibition → dsRNA → IFN + anti-PD1 → distinct clone elimination (SPLINTR); up to 188 pts	[118] NCT06975293

*Current and emerging therapeutic agents targeting RNA modification enzymes in cancer. Compounds are grouped by target and mechanism. Development stages reflect the most advanced status as of early 2026. Potency values are from the primary publications cited. Combination strategies are listed separately at the bottom. Bold text indicates compounds in clinical development. Abbreviations: α-KG, alpha-ketoglutarate; AML, acute myeloid leukaemia; DC50, half-maximal degradation concentration; DCR, disease control rate; Dmax, maximum degradation; HNSCC, head and neck squamous cell carcinoma; IC50, half-maximal inhibitory concentration; IFN, interferon; IR, intron retention; ITP, immune thrombocytopenia; NSCLC, non-small cell lung cancer; ORR, overall response rate; PDX, patient-derived xenograft; POC, proof of concept; PROTAC, proteolysis-targeting chimera; RO, transcriptional run-on; SAM, S-adenosylmethionine.

The mechanism of action linking METTL3 inhibition to anti-tumour immunity has been elucidated in detail by Mayday et al. Using STC-15 and the closely related tool compound STM3675, they demonstrated that m6A loss triggers aberrant RNA processing. Specifically, it induces intron retention (IR) and transcriptional run-on (RO) events downstream of highly m6A-modified long exons [116]. These aberrant transcripts form double-stranded RNA (dsRNA) that is recognized by the cytoplasmic sensor MDA5, activating type I interferon signalling and remodelling the tumour microenvironment towards a pro-inflammatory state [116]. This mechanism is particularly notable from a translational control perspective: METTL3 inhibition does not simply reduce m6A-dependent translation of oncogenes, but fundamentally disrupts co-transcriptional RNA processing, generating immunostimulatory substrates that convert ‘cold’ tumours into targets for immune clearance.

The clinical translation of this immunostimulatory mechanism is now being tested in combination with checkpoint blockade. In May 2025, STORM Therapeutics announced the first patient dosed in a Phase 1b/2 study (NCT06975293) evaluating STC-15 in combination with toripalimab (LOQTORZI), a next-generation PD-1 inhibitor, in patients with NSCLC, HNSCC, melanoma, and endometrial cancer [117]. The Phase 2 expansion will enrol up to 188 patients across these four tumour types. This combination is supported by the preclinical observation confirmed using SPLINTR barcoding in syngeneic mouse models that METTL3 inhibition and anti-PD1 therapy target distinct malignant clones, providing a mechanistic rationale for their non-redundant combination [118].

### The drug pipeline: from SAM-competitive inhibitors to targeted protein degradation

Beyond STC-15, a rapidly diversifying pipeline of RNA modification-targeting agents is approaching clinical readiness, spanning multiple enzymatic targets and therapeutic modalities (Table 2).

#### METTL3 inhibitors

The foundational compound STM2457, from which STC-15 was derived, demonstrated the preclinical proof of concept for METTL3 inhibition in acute myeloid leukaemia (AML). STM2457 (IC50 = 16.9 nM) selectively reduces m6A on leukaemogenic transcripts, promotes differentiation, induces apoptosis, and extends survival in multiple AML mouse models [115]. STORM Therapeutics has since disclosed next-generation compounds, including STM3006, with distinct structural features and improved potency profiles suitable for expansion into solid tumour indications [119]. Independent structure-based discovery has produced additional SAM-competitive chemotypes, including UZH1a (IC50 = 280 nM), which occupies the SAM adenosine pocket of METTL3 and reduces cellular m6A levels, albeit at micromolar concentrations owing to competition with endogenous SAM [120]. The field has also produced structurally diverse SAM-competitive scaffolds, including aminopyrimidines, pyrimidinones, and indazoles, as well as the first allosteric inhibitor, eltrombopag, which targets the METTL3-METTL14 interface [121]. This expanding chemical space increases the likelihood of identifying compounds with optimal pharmacokinetic properties for different tumour compartments and patient populations.

#### FTO demethylase inhibitors

Targeting the m6A ‘eraser’ FTO provides a complementary strategy that increases, rather than decreases, global m6A levels. FB23-2 (IC50 = 2.6 µM) demonstrates anti-proliferative activity across AML cell lines and patient-derived xenograft models [122]. R-2-hydroxyglutarate, the oncometabolite accumulating in IDH-mutant cancers, functions as a natural FTO inhibitor by competing with α-ketoglutarate, providing an elegant example of how cancer metabolism intersects with epitranscriptomic regulation [123]. Meclofenamic acid, an approved NSAID with FTO inhibitory activity, exemplifies the repurposing potential within this space [124]. From the translational control perspective, FTO inhibition is predicted to broadly enhance m6A-dependent mRNA decay (*via* the ribosome collision-degradation pathway described in ‘Integrated Modification Networks: How Multi-Layered Epitranscriptomic Crosstalk Shapes Malignant Translation’), potentially counteracting the mcm5s2U-mediated stabilization of oncogenic transcripts observed in aggressive cancers.

#### METTL3-METTL14 PROTACs

Proteolysis-targeting chimeras (PROTACs) represent a conceptual advance over catalytic inhibitors because they eliminate both the enzymatic and non-enzymatic functions of METTL3, including its scaffolding role in eIF3 recruitment and mRNA looping (see ‘Initiation Control by RNA Modifications’). WD6305, the first potent METTL3-METTL14 PROTAC (DC50 = 140 nM for METTL3, D_max_ ~92%), achieves near-complete degradation of the heterodimeric complex and suppresses AML cell proliferation more effectively than the parent inhibitor UZH2 [125]. More recently, AF151, incorporating an indole-nicotinamide chemotype structurally distinct from UZH2-based degraders, demonstrated comparable potency (DC_50_ = 430 nM) with enhanced viability inhibition and m6A reduction in AML cells [126]. The ability of PROTACs to abolish METTL3’s translation-enhancing functions, including the direct eIF3h interaction that promotes polysome formation on oncogenic mRNAs such as *BRD4* and *EGFR*, and the PABPC1-dependent mRNA circularization (described in ‘Initiation Control by RNA Modifications’), provides a mechanistic rationale for their superior anti-tumour activity compared with catalytic inhibition alone.

#### Targets beyond m6A

Although the current clinical pipeline is dominated by m6A-targeting agents, preclinical programmes are expanding to other modification axes relevant to cancer translation. NAT10, the sole ac4C writer, promotes translation of cell cycle and drug resistance genes (Elongation Control by RNA Modifications); remodelin, a NAT10 inhibitor originally developed for laminopathy, shows anti-cancer activity in preclinical models, though with limited selectivity [127]. Inhibitors of the ELP3/CTU1/2 wobble modification pathway, which tunes mRNA-tRNA modification balance at the elongation level (‘Elongation Control by RNA Modifications’ and ‘Integrated Modification Networks: How Multi-Layered Epitranscriptomic Crosstalk Shapes Malignant Translation’), remain at early discovery stages but represent compelling targets given the strong prognostic association of mcm5s2U biogenesis factors with poor outcomes in breast cancer [63].

### Combination strategies informed by translation biology

The modification networks described in ‘Integrated Modification Networks: How Multi-Layered Epitranscriptomic Crosstalk Shapes Malignant Translation’ imply that monotherapy targeting a single modification axis will face compensatory rewiring. Several emerging combination strategies are grounded in the translational biology of RNA modifications.

*METTL3 inhibition plus immune checkpoint blockade* is the most clinically advanced combination, now in Phase 1b/2 trials (NCT06975293). The mechanistic rationale is twofold: (i) METTL3 inhibition generates immunostimulatory dsRNA *via* the IR/RO pathway, creating a pro-inflammatory tumour microenvironment enriched in M1 macrophages and interferon-stimulated gene products; and (ii) METTL3 inhibition and anti-PD1 therapy eliminate distinct clonal populations, as demonstrated by SPLINTR barcoding [118]. Importantly, the IFN pathway activation induced by METTL3 inhibition upregulates MHC-I expression and antigen presentation, potentially overcoming a key resistance mechanism to checkpoint immunotherapy.

*METTL3 inhibition plus venetoclax* exploits the observation that m6A deposited by METTL3 stabilizes BCL2 mRNA translation. Pharmacological METTL3 inhibition with STC-15 synergizes with venetoclax in AML patient-derived xenograft models, with combination-treated animals showing extended survival compared with either agent alone [131]. This combination directly leverages METTL3’s role in translational enhancement of the anti-apoptotic programme.

*Pan-epitranscriptomic combination targeting* represents a forward-looking strategy derived from the mRNA-tRNA modification balance described in ‘Integrated Modification Networks: How Multi-Layered Epitranscriptomic Crosstalk Shapes Malignant Translation’. Because the ELP3/CTU1/2-installed tRNA modification mcm5s2U counteracts m6A-induced ribosome collisions and mRNA decay, simultaneous inhibition of METTL3 (reducing mRNA m6A) and ELP3 pathway disruption (reducing tRNA mcm5s2U) could, in principle, act on opposing arms of the same regulatory axis – though whether the net effect would be synergistic or antagonistic will depend on transcript-specific modification stoichiometries [63]. The pan-epitranscriptomic framework thus provides a rational basis for designing modification-informed combinations rather than empirical pairings.

### Biomarkers and the path to epitranscriptomic precision medicine

The clinical implementation of RNA modification therapeutics will require biomarkers for patient stratification, target engagement, and response monitoring. Three tiers of biomarker evidence are emerging.

*Single-enzyme prognostic biomarkers* are the most clinically mature. Elevated METTL3 expression correlates with poor prognosis in AML, colorectal cancer, and hepatocellular carcinoma across independent validation cohorts [15]. METTL1 overexpression predicts chemotherapy resistance in multiple solid tumours, consistent with its role in enhancing m7G-dependent translation of cell cycle regulators (see ‘Elongation Control by RNA Modifications’) [21]. FBL levels predict IRES-dependent translation capacity and correlate with poor outcomes in breast, prostate, and cervical cancers (see ‘Initiation Control by RNA Modifications’) [25]. While informative individually, these single-gene markers do not capture the network-level modification dynamics that underlie treatment response.

*Multi-modification network signatures* offer superior predictive power. The pan-epitranscriptomic gene expression signature developed by Linder et al. based on the relative expression of mcm5s2U biogenesis factors (ELP3, CTU1, CTU2) *versus* m6A pathway components (METTL3, METTL14, WTAP, YTHDF2) stratifies breast cancer patients by overall survival independently of conventional molecular subtypes [63]. This signature directly reflects the translational balance between mRNA m6A-mediated decay and tRNA modification-mediated stabilization, providing a mechanistically grounded biomarker that could predict response to agents perturbing either arm. Extension of this approach to other tumour types and integration with tRNA and rRNA modification status (*e.g*., m1acp3Ψ levels, SNORA24 expression) could yield comprehensive epitranscriptomic classifiers.

*Pharmacodynamic and liquid biopsy biomarkers* are essential for monitoring on-target drug activity. In the STC-15 Phase 1 trial, m6A levels in polyadenylated RNA from peripheral blood samples served as a robust pharmacodynamic readout, demonstrating rapid and sustained target engagement at all dose levels [9]. Gene expression enrichment of interferon-stimulated gene families (*IFIT*, *OAS*) in patients with longer treatment durations provided additional mechanistic confirmation. Circulating RNA modifications detectable in cell-free RNA represent an attractive, though technically challenging, frontier for non-invasive disease monitoring. As companion diagnostic assays mature, whether through RNA modification-specific immunohistochemistry, quantitative PCR panels, or mass spectrometry-based m6A quantification, the integration of these biomarkers with clinical decision-making algorithms will be critical for optimizing the therapeutic index of epitranscriptomic agents.

Linking these biomarker tiers to specific agents clarifies their potential clinical utility. Single-enzyme markers could provide a first-pass triage; for example, high *METTL3* or *METTL1* expression might nominate patients for writer-directed inhibitors, or high *FBL* expression flag tumours reliant on IRES-dependent translation. The multi-modification, pan-epitranscriptomic signature (the relative expression of mcm5s2U-pathway *versus* m6A-pathway factors) is conceptually better suited to predicting the direction of response to agents acting on opposing arms of the same axis: tumours skewed towards mcm5s2U-mediated stabilization might be preferentially sensitive to combined METTL3- and ELP3-pathway perturbation, whereas m6A-pathway-high tumours might respond differently to writer *versus* eraser inhibition. Pharmacodynamic readouts (blood m6A levels, interferon-stimulated gene induction) would then confirm target engagement and the immunostimulatory mechanism during treatment. We emphasize that this framework is hypothesis-generating: none of these biomarker-drug pairings has yet been validated prospectively, and doing so is an important priority for the field.

### Perspective: from translation biology to therapeutic strategy

The therapeutic landscape of RNA modification inhibitors is at an inflection point. STC-15 has validated the clinical tractability of the epitranscriptomic target class, and the mechanistic understanding of how METTL3 inhibition generates immunostimulatory dsRNA provides a clear biological rationale for combination with checkpoint blockade. The expanding pipeline, from next-generation SAM-competitive inhibitors through PROTACs to agents targeting FTO, NAT10, and tRNA modification pathways, reflects the breadth of therapeutic opportunities identified by the translational biology described throughout this review. Crucially, the network perspective developed in ‘Integrated Modification Networks: How Multi-Layered Epitranscriptomic Crosstalk Shapes Malignant Translation’ argues against monotherapy-centric development: the interconnected nature of mRNA, tRNA, and rRNA modification systems means that compensatory rewiring will likely limit single-agent durability. Rational, biology-informed combinations, such as pairing mRNA modification inhibitors with tRNA pathway modulators, or coupling catalytic inhibitors with targeted degraders, will be essential for durable responses. Equally, the multi-modification biomarker signatures now emerging from translational studies provide the precision medicine framework needed to match patients with optimal therapeutic strategies. The convergence of early clinical validation, mechanistic understanding, and biomarker development suggests that epitranscriptomic therapeutics may become a valuable additional class in cancer treatment, although this remains to be established in controlled trials.

## Future directions and concluding perspectives

### Technological frontiers: toward multi-modification, single-cell resolution

The mechanistic insights and therapeutic opportunities described in this review rest on a foundation of detection technologies that is evolving rapidly. Among the most promising advances is the maturation of nanopore direct RNA sequencing (DRS), which now enables simultaneous detection of m6A, m5C, pseudouridine, and inosine on individual native RNA molecules using Oxford Nanopore’s RNA004 chemistry and deep-learning basecallers such as Dorado and Dorado2 [57]. When combined with single-molecule co-modification frameworks like CHEUI [54] and transfer-learning platforms such as TandemMod [132], these tools are beginning to deliver the multi-modification, single-molecule profiling that the field has long required. Application of these technologies to primary tumour samples, particularly when coupled with emerging single-cell epitranscriptomic approaches [133], will be essential for resolving intra-tumour heterogeneity in modification landscapes and understanding how distinct cellular subpopulations within a tumour exploit different epitranscriptomic programs to sustain malignant translation. We reiterate, however, that the single-molecule co-occurrence analyses on which parts of the Integrated Modification Networks section rely remain dependent on these still-maturing computational models, and their quantitative interpretation should be treated with corresponding caution.

A complementary frontier is the development of circulating RNA modification biomarkers for liquid biopsy applications. The recent demonstration that nanopore-based profiling of 2′-O-methylation on a circulating rRNA fragment can discriminate lung cancer patients from controls (AUC = 0.84) provides proof of principle that epitranscriptomic signatures in cell-free RNA are clinically accessible [134]. Extending this approach to multi-modification panels encompassing m6A, m5C, and Ψ on circulating RNA species could yield non-invasive diagnostic and pharmacodynamic tools that complement the tissue-based biomarkers discussed in ‘Therapeutic Targeting of RNA Modifications in Cancer Translation’.

### Expanding the target space: A-to-I editing and beyond

This review has focused on modifications that directly regulate translation and considered m6A, m5C, pseudouridine, 2′-O-methylation, m7G, ac4C, and wobble uridine modifications, but the epitranscriptomic landscape extends further. Adenosine-to-inosine (A-to-I) RNA editing, catalysed by ADAR1, represents a mechanistically distinct modification axis with profound implications for cancer immunity and translation. ADAR1-mediated editing of endogenous double-stranded RNAs prevents their recognition by cytoplasmic sensors MDA5 and PKR, thereby suppressing type I interferon signalling and the PKR-mediated translational shutdown that would otherwise limit tumour growth [135]. The parallels with METTL3 inhibition which also generates immunostimulatory dsRNA, albeit through a different mechanism (Therapeutic Targeting of RNA Modifications in Cancer Translation), are striking, and small-molecule ADAR1 inhibitors, including ZYS-1, are now demonstrating anti-tumour efficacy and synergy with checkpoint blockade in preclinical prostate cancer models [129]. A first ADAR1 inhibitor, ADAR1i-124, has recently shown capacity to activate both MDA5 and ZBP1 pathways and to synergize with DNA methyltransferase inhibitors in re-sensitizing refractory cancer cells [130]. Because A-to-I editing directly regulates PKR-dependent translational control and modulates the immunogenicity of tumour-derived transcripts, incorporating this axis into the translational framework developed here will be important for understanding the full scope of epitranscriptomic regulation in cancer.

Other underexplored frontiers include the role of RNA modifications in drug-tolerant persister cells (DTPs), which are transient, phenotypically plastic cellular subpopulations that survive initial therapy and seed relapse. Emerging evidence indicates that m6A and other RNA methylations regulate the post-transcriptional programmes that sustain DTP survival, representing a critical link between epitranscriptomic plasticity and acquired drug resistance [136]. Understanding how translational reprogramming through RNA modifications enables the DTP state may reveal new strategies for preventing therapy-resistant relapse.

### Limitations, reproducibility and unresolved questions

Several fundamental questions remain. First, the ‘modification code’, which encompasses the rules governing how combinations of modifications on the same transcript, or across mRNA-tRNA-rRNA axes, collectively determine translational output, is largely undeciphered. The pan-epitranscriptomic framework of Linder et al. [63] provides a powerful conceptual model for the m6A-mcm5s2U pair, but extending this to the full combinatorial space of > 170 modifications will require systematic perturbation studies paired with multi-modification profiling. Second, the relationship between modification stoichiometry and functional outcome remains poorly defined: at most modified sites, only a fraction of transcript copies carry the modification, yet the biological significance of this heterogeneity is rarely considered in cancer studies. Third, the contribution of mitochondrial RNA modifications to cancer translation glimpsed through NSUN3/ALKBH1 (see ‘Initiation Control by RNA Modifications’) represents a largely uncharted territory with therapeutic potential, particularly for anti-metastatic strategies targeting metabolically defined tumour-initiating cells.

Beyond these mechanistic questions, several methodological and interpretive limitations temper the conclusions that can currently be drawn. First, much of the cancer epitranscriptomics literature is correlative: associations between modifier expression and clinical outcome are abundant, but causal, transcript-resolved evidence that a specific modification drives a specific translational change is available for relatively few, well-studied cases. Second, the detection technologies on which the field depends carry significant caveats (see ‘The Epitranscriptomic Toolkit’): antibody-based mapping such as MeRIP-seq suffers from limited resolution and reproducibility, while nanopore direct RNA sequencing, although increasingly powerful, remains dependent on supervised computational models whose accuracy varies by modification type and has not been comprehensively benchmarked, particularly for stoichiometry and for co-occurring modifications. Third, modification stoichiometry is rarely measured yet mechanistically central: because most sites are sub-stoichiometric, population-averaged measurements can obscure the behaviour of the modified subpopulation that actually carries the regulatory signal. Fourth, the mechanistic links to translation are far better established for m6A than for m5C, ac4C, m1A and A-to-I editing, some of whose mRNA-level maps have themselves been contested on technical grounds; we have therefore treated mechanistic claims for these marks more conservatively. Finally, the integrated-network and ‘specialized ribosome’ models discussed in this review, while heuristically valuable, remain largely conceptual and await direct, simultaneous, single-molecule validation in primary tumours, and the therapeutic programmes built on them are still at an early clinical stage. Progress on each of these fronts, including causal perturbation, orthogonal and standardized detection, stoichiometry-aware analysis, and prospective clinical validation, will determine how much of the framework presented here ultimately translates into patient benefit.

## Conclusions

The field of epitranscriptomic translational control in cancer has matured from descriptive associations to mechanistic understanding and clinical translation within a remarkably compressed timeframe. The translation-centric framework adopted in this review reveals several overarching principles. First, RNA modifications do not merely fine-tune translation; they fundamentally redirect it, switching between cap-dependent and IRES-dependent initiation, coupling elongation dynamics to mRNA decay, and converting stop codons into sense codons. Second, the functional impact of any single modification depends on its molecular context: the modification landscape of the same transcript, the tRNA modifications that decode it, the rRNA modifications of the ribosomes that translate it, and the chromatin signals that directed its initial deposition. Third, this network architecture has direct therapeutic implications: monotherapy targeting a single modification node will face compensatory rewiring, arguing for rational, biology-informed combination strategies.

The clinical validation of METTL3 inhibition with STC-15 and its mechanistic convergence with emerging ADAR1 inhibitors through shared dsRNA-sensing pathways establishes epitranscriptomic enzymes as a tractable and expanding therapeutic class. The multi-modification biomarker signatures now emerging from translational studies provide the precision medicine framework needed to match patients with optimal strategies. As detection technologies mature to enable multi-modification, single-molecule, single-cell profiling of clinical tumour samples, and as the drug pipeline expands beyond m6A to encompass tRNA, rRNA, and A-to-I editing targets, the integration of epitranscriptomic biology with translational oncology promises to yield a new generation of cancer therapeutics grounded in the molecular logic of protein synthesis.

## Data Availability

No new data were created or analysed in this study. Data sharing is not applicable to this review article.
